# Non-Invasive Malaria Detection in Sub-Saharan Africa Using a DNA-Based Sensor System

**DOI:** 10.3390/s24247947

**Published:** 2024-12-12

**Authors:** Trine Juul-Kristensen, Celine Thiesen, Line Wulff Haurum, Josephine Geertsen Keller, Romeo Wenceslas Lendamba, Rella Zoleko Manego, Madeleine Eunice Betouke Ongwe, Birgitta Ruth Knudsen, Eduardo Pareja, Eduardo Pareja-Tobes, Rodrigo Labouriau, Ghyslain Mombo-Ngoma, Cinzia Tesauro

**Affiliations:** 1 Department of Molecular Biology and Genetics, Universitetsbyen 81, Aarhus University, 8000 Aarhus, Denmark; tjk@mbg.au.dk (T.J.-K.); ceth@biomed.au.dk (C.T.); lwh@mbg.au.dk (L.W.H.); jgk@mbg.au.dk (J.G.K.); brk@mbg.au.dk (B.R.K.); 2 Department of Biomedicine, Høegh-Guldbergs Gade 10, Aarhus University, 8000 Aarhus, Denmark; 3 Centre de Recherches Médicales de Lambaréné (CERMEL), Lambaréné BP 242, Gabon; wenceslas.lendamba@cermel.org (R.W.L.); rella.zoleko@cermel.org (R.Z.M.); m.e.betouke_ongwe@lumc.nl (M.E.B.O.); ghyslain.mombongoma@cermel.org (G.M.-N.); 4 Department of Implementation Research, Bernhard Nocht Institute for Tropical Medicine, & I. Dep. of Medicine, University Medical Centre Hamburg-Eppendorf, Bernhard-Nocht-Strasse 74, 20359 Hamburg, Germany; 5 Institut de Recherche en Ecologie Tropicale, CENAREST, Libreville BP 13354, Gabon; 6 Nisonide SL, 18008 Granada, Spain; eduardo.pareja@nisolab.com (E.P.); edu@nisolab.com (E.P.-T.); 7 Department of Mathematics, Aarhus University, Ny Munkegade 118, 8000 Aarhus, Denmark; rodrigo.labouriau@math.au.dk

**Keywords:** malaria, diagnosis, rolling circle amplification, saliva, topoisomerase 1

## Abstract

Malaria poses a serious global health problem, with half the world population being at risk. Regular screening is crucial for breaking the transmission cycle and combatting the disease spreading. However, current diagnostic tools relying on blood samples face challenges in many malaria-epidemic areas. In the present study, we demonstrate the detection of the malaria-causing *Plasmodium* parasite in non-invasive saliva samples (N = 61) from infected individuals by combining a DNA-based Rolling-circle-Enhanced-Enzyme-Activity-Detection (REEAD) sensor system with a chemiluminescence readout that could be detected with an in-house-developed affordable and battery-powered portable reader. We successfully transferred the technology to sub-Saharan Africa, where the malaria burden is high, and demonstrated a proof of concept in a small study (N = 40) showing significant differences (*p* < 0.00001) between malaria-positive individuals (N = 33) and presumed asymptomatic negative individuals (N = 7) all collected in Gabon. This is the first successful application of the REEAD sensor system for the detection of malaria in saliva in a high-epidemic area and holds promise for the potential future use of REEAD for malaria diagnosis or surveillance based on non-invasive specimens in sub-Saharan Africa.

## 1. Introduction

Malaria is among the most serious global health issues. It is a major cause of death and illness in many low- and middle-income countries with an estimated 249 million cases and 608,000 deaths in 2022, particularly among young children and pregnant women [1]. Today, nearly half of the world population is at risk of malaria. These numbers may even increase with climate change that alters the geographic distribution of malaria by altering the habitats suitable for malaria-parasite-carrying mosquitos [2,3,4]. This development underlines the importance of regular screening in populations at risk of infection and in areas where malaria is currently under control. The World Health Organization (WHO) has set ambitious goals for malaria elimination in 26 countries by 2025 [5], bringing the number of certified malaria-free countries to 69 [6]. Screening and treating asymptomatic carriers allow us to break the transmission cycle and are vital steps toward these targets. Moreover, regular screening and surveillance of malaria can provide valuable data on how climate change impacts the pattern of malaria transmission.

Malaria is caused by infections with *Plasmodium* parasites, which typically reside in red blood cells [7,8]. Consequently, malaria is currently diagnosed in blood. The gold standards are light microscopy of thick or thin blood smears or polymerase chain reaction (PCR) [9,10]. These methods are often either unavailable or limited in low-resource settings where malaria is prevalent. Here, antigen rapid diagnostic tests (RDTs) are commonly used. However, the sensitivity of RDTs is low for patients with low-level parasitemia, and they fail to detect all malaria-causing *Plasmodium* species [11]. Due to these limitations and cultural reluctance to give blood in many malaria-endemic areas, new diagnostic tools allowing for sensitive and specific detection of all malaria-causing *Plasmodium* species in non-invasive samples at low-resource settings are highly needed [12].

**Figure 1:**
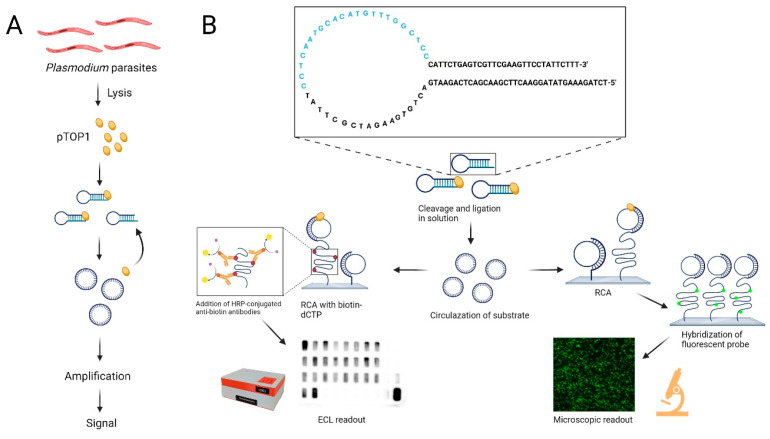
The REEAD sensor system. (**A**) The advantage of using pTOP1 as a biomarker for the detection of *Plasmodium* infections. Each parasite contains a high number of pTOP1 enzymes that each generate multiple DNA products without being consumed in the process. (**B**) The top panel shows the sequence and structure of the pTOP1-specific DNA substrate with the primer annealing site shown in blue. Cleavage–ligation by pTOP1 converts the substrate to a closed circle that is hybridized to a glass slide and amplified by RCA in the presence of (i) dNTPs with biotin-conjugated dCTPs for chemiluminescence readout (**left lower panel**) or (ii) without modified dNTPs followed by hybridization to fluorescently labeled probes for readout in a fluorescence microscope (**right lower panel**).

DNA sensors or sensor systems composed of DNA may present attractive solutions to this challenge. DNA sensors for the sensitive and specific detection of biomarkers benefit markedly from advances of this century in the chemical synthesis of modified DNA oligonucleotides. During the past 15+ years, a large number of DNA sensors or sensor systems for the detection of different biomarkers, including small molecules [13,14,15,16], proteins [17,18], or enzyme activities [18,19,20,21,22,23,24,25,26], have been described. Besides the ease by which the secondary structure and functionality of DNA molecules can be manipulated for the detection of specific biomarkers [25,27,28], DNA can be amplified by polymerases, making it an excellent material for detection systems with enhanced sensitivity [29,30,31]. DNA sensor systems that rely on isothermal amplification systems such as rolling circle amplification (RCA) catalyzed by highly processive polymerases such as phi29 polymerase offer the additional advantage of being directly quantitative. When employing an enzyme activity as a biomarker for detection, the sensitivity is further increased as each enzyme generates many products that can each be amplified before detection without being consumed by the process (see schematic outline in Figure 1A).

By taking advantage of linear DNA substrates that could be converted to a closed DNA circle and act as a template for phi29-mediated amplification only by their specific target enzyme, we previously reported Rolling-circle-Enzyme-Enhanced-Amplification-Detection (REEAD) DNA sensor systems for detecting disease-causing human pathogens such as *Plasmodium*, *Mycobacteria*, and HIV [26,32,33,34,35]. For the detection of *Plasmodium*, we utilized a single-stranded DNA substrate that folds into a hairpin structure with a primer annealing sequence in the single-stranded loop region and a recognition site that can be cleaved and ligated specifically by the life-essential *Plasmodium*-expressed enzyme topoisomerase1 (pTOP1) [36,37]. As illustrated in Figure 1B, pTOP1 converts the substrate to a single-stranded DNA circle that can function as a template for RCA in a solid support format or solution. Detection can be achieved by fluorescence labeling or coupling with Horse Radish Peroxidase (HRP), which enables different readouts [38]. Using this setup, we have demonstrated the specific detection of all human malaria-causing *Plasmodium* species in blood and saliva with estimated detection limits of 0.06 and 2 parasites/µL, respectively [39]. However, all previous testing of the malaria-specific REEAD was performed in high-resource laboratory facilities using samples from confirmed positives collected in sub-Saharan Africa or Asia that were tested against negative samples collected in Denmark [33,39]. Moreover, all previous tests relied on a rather complicated microfluidics-based extraction system to release active enzymes from the *Plasmodium* parasites, while a simple colorimetric readout was applied to a few tests with promising results [33,39].

In the present study, we demonstrate the detection of *Plasmodium* parasites in saliva from confirmed infected individuals in a malaria clinic in Gabon. This was achieved using an extraction method combined with a chemiluminescent-based readout for enhanced detection. For readout, we present an in-house built battery-driven affordable 3D printed portable reader optimized for the detection of chemiluminescence REEAD signals. Using 3D printing enables the rapid, cost-effective manufacturing of lightweight devices, making it ideal for low-resource settings by addressing affordability and logistical challenges, including ease of transport and deployment in remote areas. Using this reader for testing saliva for the presence of *Plasmodium* parasites in Lambaréné, Gabon, we demonstrate significant (*p* < 0.00001) differences between a population of confirmed malaria-positive (N = 33) and a population of presumed asymptomatic negative individuals (N = 7) all locally collected in Gabon. These results hold promise for the applicability of the malaria-specific REEAD in sub-Saharan Africa where the reported detection of *Plasmodium* parasites in saliva may also be relevant for surveillance programs.

## 2. Materials and Methods

### 2.1. Reagents

All chemicals were purchased from Sigma Aldrich, Søborg, Denmark. CodeLink Activated HD slides (#DHD1-0023) were from SurModics (Saint Paul, MN, USA), Pertex glue (#00801) was from Histolab (Askim, Sweden), a PAP Pen was from Super HT (Japan), a silicone Wellmaker grid was custom-made by Grace bio-labs (Bend, OR, USA), Vectashield without DAPI (#H-1000) was from Vector Laboratories (Burlington, ON, Canada), exonuclease I and III were from Thermo Scientific (Roskilde, Denmark), and glass beads 150–212 μm (#70-100 U.S. sieve) were from Sigma Aldrich (Steinheim, Germany).

### 2.2. DNA Oligonucleotides

DNA oligonucleotides were synthesized by Sigma Aldrich, Søborg, Denmark. The sequences were as follows:

- 5′-Amine REEAD primer:
  5′-[AmC6] CCAACCAACCAACCAAGGAGCCAAACATGTGCATTGAGG-3′;
- pTOP1substrate:
  5′-TCTAGAAAGTATAGGAACTTCGAACGACTCAGAATGACTGTGAAGA TCGCTTATCCTCAATGCACATGTTTGGCTCCCATTCTGAGTCGTTCGAAGTTCCTATTCTTT-3′;
- hTOP1substrate:
  5′-AGAAAAATTTTTAAAAAAACTGTGAAGATCGCTTATTTTTTTAAAAAT TTTTCTAAGTCTTTTAGATCCCTCAATGCACATGTTTGGCTCCGATCTAAAAGACTTAGA-3′;
- Fluorescent probe:
  5′-[FAM] CCT CAA TGC ACA TGT TTG GCT CC-3′.

### 2.3. Saliva and Blood Samples from Malaria Patients and Uninfected Individuals

Saliva samples from patients diagnosed with malaria were obtained at the CERMEL, Albert Schweitzer Hospital, Lambaréné, Gabon [40]. The patients were diagnosed first using a rapid diagnostic test (RDT) in matching blood, followed by thick smear microscopy. The study was conducted in accordance with the Declaration of Helsinki, and samples were collected and analyzed following local regulations and guidelines. Patients’ samples were obtained from the malaria screening activities conducted at CERMEL according to ethical clearance granted for ongoing studies and informed consent provided by each individual or their representatives. Thick smear microscopy was performed by two independent readers using the Lambaréné method [41]. A third reading was performed if required (e.g., if the first and second readings differed more than 50% of parasite count or if the same slide was read positive/negative by different readers). The number of counted parasites per µL blood was recorded. The final parasitemia was determined as the average of the first and second readings (or, if a third reading was performed, the average of the two closest results). In the case of testing by RDTs, the “Paracheck Pf” test was used.

### 2.4. REEAD

#### 2.4.1. Preparation of Slides

A CodeLink Activated HD slide was cut into an appropriate size. The slide was either attached to an object glass by Pertex glue followed by drawing squared-shaped fields with a Mini PAP Pen (for the fluorescence microscope readout) or attached to a custom-made silicone grid to create delimited rectangular-shaped wells of 1.2 mm × 2.8 mm dimensions termed the Wellmaker. Then, 10 µM of the 5′-Amine REEAD primer in 300 mM Na_3_PO_4_ pH 8 was coupled to the slide. The slide was incubated overnight in a humidity chamber with saturated NaCl. The slide was subsequently blocked in blocking buffer (50 mM Tris, 50 mM Tris-HCl, and 50 mM ethanolamine, pH 9) for 30 min at 50 °C and washed twice in ddH_2_O before it was washed in wash buffer 1 (4× SSC and 0.1% SDS) for 30 min at 50 °C and finally washed twice in ddH_2_O.

#### 2.4.2. Extraction of Saliva Samples

Frozen saliva samples were thawed on ice. Subsequently, 200 µL of the saliva was homogenized by passing through a 0.5 mL tube with a hole in the bottom by centrifugation at 1000× *g* for 3 min. The homogenized saliva was collected in a 1.5 mL tube containing 25 mM HEPES pH 7.9, 30 mM NaCl, 0.02% Triton x-100, and 1 mM DTT. The samples were incubated for 15 min on ice before the addition of glass beads (1:1 vol/vol). The samples were vortexed using an automated vortex (Scientific Industries #SI-D258) for 30 s with a 1 min break on ice. Vortexing was repeated 2–5 times as described.

#### 2.4.3. Circularization with Saliva Samples

Circularization of the pTOP1 substrate was carried out by incubating 4 µL of the extracted saliva with 0.5 µM of the substrate in the presence of a buffer containing 250 mM NaCl, 10 mM Tris-HCl pH 7.5, and 1 mM EDTA for 1 h at 37 °C in a total volume of 20 µL. The reaction was stopped by heat inactivation at 95 °C for 5 min. The circles were hybridized to the 5′-Amine REEAD primer coupled slides overnight in a humidity chamber at room temperature and subsequently washed in 100 mM Tris-HCl pH 7.5, 150 mM NaCl, 0.3% SDS for 1 min, followed by washing in 100 mM Tris-HCl pH 7.5, 150 mM NaCl, 0.05% Tween20 for 1 min, and finally dehydrated for 1 min in 70% EtOH.

#### 2.4.4. Circularization with *P. falciparum* TOP1 Spiked in Saliva

Saliva was extracted as described above and mixed with purified pfTOP1 (different concentrations as indicated) before being added into a buffer containing 250 mM NaCl, 10 mM Tris-HCl pH 7.5, and 1 mM EDTA. The circularization reaction with the pTOP1 substrate incubated was carried out for 1 h at 37 °C and subsequently stopped by heat inactivation at 95 °C. The circles were hybridized to the 5′-Amine REEAD primer coupled slides as described above.

#### 2.4.5. Circularization with Purified *P. falciparum* TOP1 and Human TOP1

Circularization of the substrate with either pfTOP1 or hTOP1 substrate was carried out by incubating 1 ng/µL pfTOP1 or 120 ng/µL hTOP1 with 0.5 µM of the specific substrate in the presence of a buffer containing 10 mM Tris-HCl pH 7.5 and 5 mM EDTA and supplemented with 250 mM NaCl (pfTOP1) or 50 mM NaCl (hTOP1) for 1 h at 37 °C. The reaction was stopped either by heat inactivation at 95 °C for 5 min (pfTOP1) or by adding NaCl to a final concentration of 250 mM (hTOP1). The circles were hybridized to the 5′-Amine REEAD primer coupled slides as described above.

Before hybridizing some of the circles, the circularization product was digested with 40 units of exonuclease III and 4 units of exonuclease I for 1 h at 37 °C. Exonuclease digestion was heat inactivated at 95 °C for 5 min.

#### 2.4.6. Rolling Circle Amplification and Detection Using Fluorescence Microscope

The rolling circle amplification reaction was performed in 1× Phi29 buffer (50 mM Tris-HCl pH 7.5, 10 mM MgCl_2_, 10 mM (NH_4_)_2_SO_4_, 4 mM DTT) supplemented with 0.2 µg BSA, 1 mM dNTP, and 1 unit of Phi29 polymerase. The reaction was carried out for 1 h at 37 °C in a humidity chamber, followed by washing as previously described. The rolling circle products were detected by hybridizing 2 µM of the fluorescent probe in a buffer containing 2× SSC, 20% formamide, and 5% glycerol for 30 min in a humidity chamber at 37 °C. The slides were washed as previously described, mounted with Vectashield, and covered with cover glass. The slides were analyzed in an Olympus IX73 fluorescence microscope with a 60× objective. Twelve images of the fluorescent signals were acquired for each sample and quantified using Image J.

#### 2.4.7. Rolling Circle Amplification and Detection Using CCD Camera or VPCIReader

Rolling circle amplification was performed in 1× Phi29 buffer (50 mM Tris-HCl pH 7.5, 10 mM MgCl_2_, 10 mM (NH_4_)_2_SO_4_, 4 mM DTT) supplemented with 0.2 µg BSA, 100 µM dATP, 100 µM dTTP, 100 µM dGTP, 90 µM dCTP, 10 µM biotin-dCTP, and 1 unit Phi29 polymerase. The reaction was carried out for 2 h in a humidity chamber at 37 °C. The Wellmaker was then washed as previously described for the microscope slide. Subsequently, HRP-conjugated anti-biotin antibody was diluted 1:300 in a buffer containing 1x TBST (20 mM Tris-HCl pH 9, 150 mM NaCl, 0.05% Tween20 pH 9) supplemented with 5% skimmed dry milk, and 5% BSA and added to the wells of the Wellmaker for 1 h at room temperature. The Wellmaker was washed 3 × 5 min in 1x TBST, and finally, 2 µL of 1:1 ECL mixture was added to allow chemiluminescence readout using a CCD camera or the VPCIReader.

#### 2.4.8. VPCIReader Usage

The VPCIReader is operated through a web application. Upon connecting to the VPCIReader, the device undergoes calibration to a tray that does not contain a slide, utilizing a dark frame image for this process. Following calibration, the slide is inserted into the plate assemblers located within the tray. With the slide placed in the VPCIReader, the light intensity emitted by each sample can be measured using the web application. The VPCIReader delivers an image of the slide along with a corresponding table that presents quantitative values of light intensity. For a comprehensive overview of the usage, see Supplementary Materials S2.

### 2.5. Protein Purifications

#### 2.5.1. *P. falciparum* TOP1

Purified as previously described [39].

#### 2.5.2. Human TOP1

Purified as previously described [42].

#### 2.5.3. Phi29 Polymerase

Purified as previously described [42].

### 2.6. Statistical Analysis

For statistical description, see Supplementary Materials (S1A and S1B).

## 3. Results and Discussion

### 3.1. Semiquantitative Detection of the Plasmodium-SPECIFIC Biomarker pTOP1 by the Use of Chemiluminescence Readout

In the original pTOP1-specific REEAD setup, the hybridization of fluorescently labeled probes to the generated RCA products allowed for the visualization of each product as a fluorescent spot in a fluorescence microscope [33]. As each RCA product corresponded to one circle generated by one pTOP1 cleavage–ligation event, the assay was directly quantitative, allowing for measurement of pTOP1 activity at the single-catalytic-event level. This readout is highly sensitive but tedious to perform and requires a rather sophisticated fluorescence microscope. Hence, it is unsuited for diagnostic purposes in low-resource settings. More suitable for user-friendly detection in rural areas, we here demonstrate semiquantitative detection of pTOP1 using a chemiluminescent readout and describe the design and construction of a portable reader for quantitative detection of signals.

**Figure 2:**
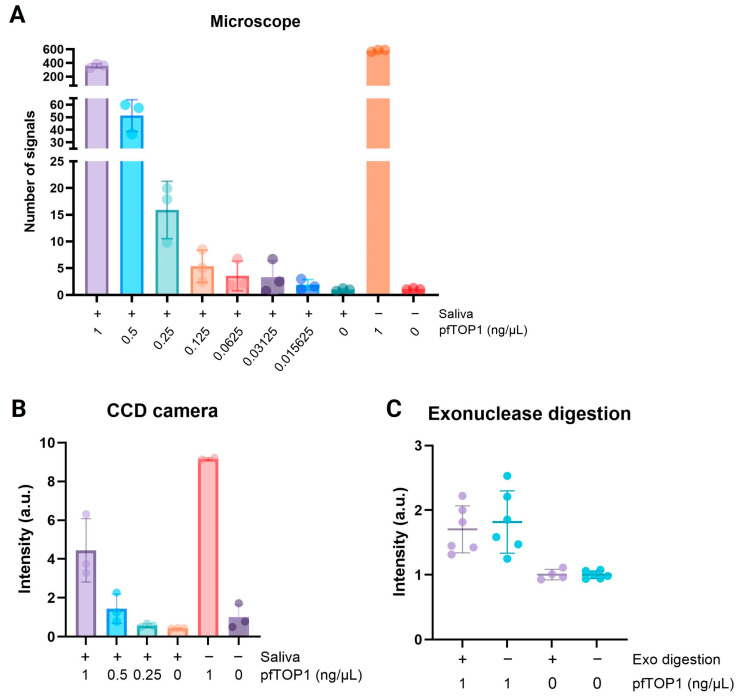
Detection of pfTOP1 with chemiluminescence REEAD. (**A**,**B**) Bar charts showing the results of analyzing 1, 0.5, 0.25, 0.125, 0.0625, 0.03125 ng/µL pfTOP1 spiked in saliva by REEAD using the fluorescence readout detected using a microscope (**A**) or the chemiluminescence readout detected using a commercial CCD camera (**B**). As controls, samples without pfTOP1 (one with and one without saliva) were included. As a positive control, a sample with 1 ng/µL pfTOP1 without saliva was used. The identity of the samples is indicated below the bar charts. The experiments were performed in triplicates (indicated by each dot). To compensate for slide-to-slide variations, the signals obtained by either the microscope or chemiluminescence readout were normalized to the average of the samples with saliva and 0 ng/µL pfTOP1 and plotted as mean +/− standard deviation (SD). (**C**) Graphic depiction of the results obtained when testing the effect of removing unreacted DNA substrate before chemiluminescence readout. The identity of the samples is shown below the graph. Each experiment was repeated four to six times (indicated by dots). To compensate for slide-to-slide variations, the chemiluminescence REEAD signals were normalized to the average intensity of samples without pfTOP1 without exonuclease digestion (Exo) and plotted as mean +/− SD.

To enable a chemiluminescent readout, biotin-conjugated nucleotides were incorporated in the RCA products. This allowed for the binding of HRP-conjugated anti-biotin antibodies and subsequent chemiluminescent visualization of the products upon HRP-catalyzed conversion of luminol to 3-aminophthalate that emits light at 425 nm (see Figure 1B). This protocol was previously used to demonstrate semiquantitative detection of human Topoisomerase 1 (hTOP1) activities with a detection limit approximately 10x higher than the detection limit observed in a fluorescence microscope [43]. In the present study, the detection of purified recombinant pTOP1 using chemiluminescence versus fluorescence REEAD was investigated. To mimic clinical specimens, pTOP1 was diluted in saliva from uninfected individuals as described in the materials and methods section and incubated with the pTOP1-specific REEAD substrate in concentrations ranging from 1 ng/µL to 0.015625 ng/µL. The results of the chemiluminescence or fluorescence readout of the same samples are shown in Figure 2A,B. As evident from Figure 2A,B, the detection limit of the ECL readout is approximately 10x higher than the detection limit of the microscopic readout. This is similar to what we observed for purified hTOP1 [43].

Moreover, comparing the chemiluminescence or microscope reading of signals obtained with the same concentration of pTOP1 in a reaction mixture with or without added saliva demonstrated that the addition of saliva reduced the intensity of the obtained REEAD signals by approximately 50%. As the reaction steps succeeding the circularization reaction only contained trace amounts of saliva due to repeated dilutions, the reduced REEAD signals were most probably the result of inhibition of the pTOP1 cleavage–ligation reaction. Although the REEAD signals will increase with increasing pTOP1 concentrations, the inhibitory effect of the sample specimen, saliva, poses a limitation to the sample volume that can be added to the circle reaction mixture. We restricted the addition of the saliva to reaction mixtures to 20% vol/vol based on the effect observed in the current experiment where 20% vol/vol saliva was added to the circle reactions where indicated (Figure 2A,B).

Detection of pTOP1 by REEAD is based on the principle that a signal is only generated when the pTOP1-specific linear DNA substrate is converted to a circle and amplified by RCA as illustrated in Figure 1. The chemiluminescence readout is based on the labeling of RCA products by biotins via the incorporation of biotin-conjugated dCTPs each time the polymerase encounters a Guanine in the template. As evident from Figure 1, Phi29 elongation from the primer annealing site of the pTOP1 substrate to the 5′-end of the unreacted substrate will result in the incorporation of 12 biotin-conjugated dCTPs in a truncated amplification product. In principle, this can allow the binding of an HRP-conjugated anti-biotin antibody and result in chemiluminescence background signals. Such potential background signals could be avoided by removing unreacted pTOP1 substrate by exonuclease digestion before RCA. To investigate if the incorporation of biotin-conjugated dCTPs in truncated amplification products templated by unreacted substrate resulted in unspecific background signals high enough to create a problem for malaria detection, we investigated the effect of exonuclease digestion of the circles before adding Phi29 polymerase. This treatment removes un-circularized substrate and is expected to eliminate potential background signals. Figure 2C shows the results of subjecting a DNA circle mixture generated by 1 ng/µL of pTOP1 (Figure 2B, light purple bar) to RCA and chemiluminescence readout with or without exonuclease digestion. As evident from the figure, we observed no difference between the two samples. Likewise, a negative control sample without pTOP1 resulted in a minimal background signal, which was not affected by exonuclease digestion. For simplicity, we therefore decided to continue our studies using a protocol that does not involve exonuclease digestion before RCA.

### 3.2. Detection of Plasmodium in Saliva from Malaria-Positive Individuals in High-Resource Laboratory Settings

**Figure 3:**
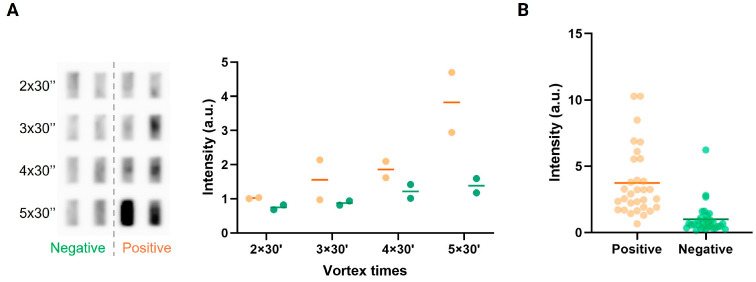
Detection of *Plasmodium* in clinical saliva samples. (**A**) (**Left panel**) Graphical depiction of chemiluminescence REEAD results obtained when measuring extracts from two saliva samples from confirmed malaria positives and two saliva samples from presumed negative individuals prepared by 2–5 vortex repetitions with glass beads. The average of the results from two individual experiments is shown by horizontal lines. (**Right panel**) Raw data obtained with a CCD camera. (**B**) The results were obtained by analyzing 30 saliva samples from confirmed malaria-positive individuals and 31 saliva samples from presumed malaria negatives using chemiluminescence REEAD. The average of the results is shown by horizontal lines. Statistics are shown in Supplementary Materials S1A. To compensate for slide-to-slide variations, the chemiluminescence REEAD signals were normalized to the average of the signals obtained by analyzing negative samples vortexed 2 times (**A**) or to the average of the signals obtained by analyzing negative samples (**B**).

Detection of *Plasmodium* parasites in clinical samples using the pTOP1-specific REEAD depends on the release of active enzymes from the parasites in a sample preparation step. We previously demonstrated effective lysis of parasites and circle generation using a droplet microfluidic setup [39]. However, even though this procedure could be performed with a simplified handheld device [33], it was difficult to handle, required extensive training, and was not suitable for routine testing of many samples. In the present study, we investigated the possibilities of releasing active enzymes from *Plasmodium* using a simple vortex protocol that is easy to perform and suitable for testing several samples simultaneously in modestly equipped test laboratories. Saliva from symptomatic malaria-positive individuals (confirmed by RDT in matching blood collected in Gabon) and from asymptomatic malaria-negative individuals collected in Denmark was vortexed repeatedly with glass beads as described in the materials and methods. The resulting lysates were mixed with the pTOP1 substrate to generate DNA circles that were subsequently amplified by RCA and detected using chemiluminescent readout. As evident from Figure 3A, two vortex repetitions of the samples did not result in any detectable difference in signals obtained from positive versus negative samples, probably due to insufficient enzyme extraction; some difference could be observed after three to four vortex repetitions, but only after five vortex repetitions was a clear difference between the positive and negative samples evident. Increasing the number of vortex repetitions did not add to signals obtained in positive samples, and seven vortex repetitions even decreased the obtained REEAD signal. We therefore continued the studies using five vortex repetitions for 30 s. Note that due to difficulties in obtaining sufficient sample volumes from the same individual, the experiment was only repeated twice. Also, due to the lack of sufficient sample volume, experiments investigating more vortex treatments were performed on samples from other patients and are, therefore, not included in the graph shown in Figure 3A. It has been a matter of debate if saliva from malaria-positive individuals contains intact *Plasmodium* parasites. Traces of the parasite, including DNA and proteins, have been observed by us and others [33,39,44], but it has been unclear if these components were remnants of already lysed parasites or if they resided inside intact parasites present in the saliva. The necessity of five vortex repetitions with glass beads before pTOP1 activity could be detected, as demonstrated in the present study, strongly argues for the presence of intact *Plasmodium* parasites in saliva from malaria-positive individuals. Our studies do, however, not provide any information on the viability of such parasites.

To address if the procedure including extraction of pTOP1 by repeated vortex combined with chemiluminescence REEAD detection could distinguish between malaria-positive and negative individuals in saliva specimens, we tested 30 saliva samples collected in Gabon from confirmed malaria-positive individuals (based on RDT analysis in matching blood) in comparison to 31 saliva samples from asymptomatic presumed malaria negative individuals collected in Denmark. The results were detected using a commercial CCD camera and the signal intensity was plotted as a function of malaria status, as shown in Figure 3B. As evident from the plot, most of the samples from malaria-positive individuals resulted in signals higher than signals obtained from samples from malaria-negative individuals, and the two populations of samples were significantly different (*p* < 0.0001) as demonstrated by Kruskal–Wallis test (see Supplementary Materials S1A). These results support the applicability of the protocol for the detection of malaria in saliva. Approximately three false positives and three false negatives could be observed. The false negatives could be the result of long-time storage and transportation of the samples collected in Gabon and/or insufficient extraction of pTOP1 from these samples. The molecular background for the false positive samples is less clear and requires further investigation.

### 3.3. Development and Proof-of-Concept Testing of an Affordable Portable Reader for Measuring Chemiluminescence REEAD Signals

**Figure 4:**
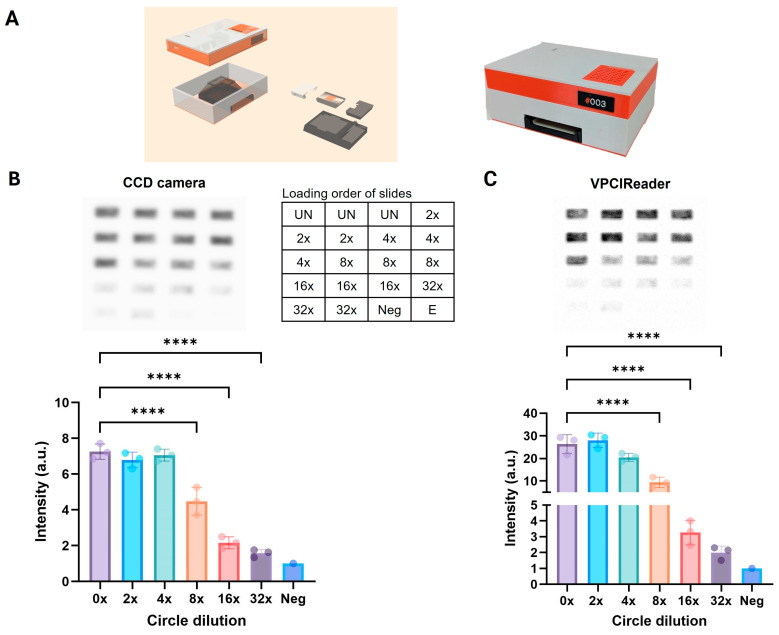
Comparison of CCD camera and portable chemiluminescence reader (VPCIReader). (**A**) Left panel, schematic showing the construction of the chemiluminescence VPCIReader. Right panel, photo of the VPCIReader. (**B**,**C**) Bar charts showing the results of analyzing titrations of test DNA circles (diluted 0 to 32 times and indicated) by capturing the results of chemiluminescence REEAD by a commercially available CCD camera (**B**) or by the developed chemiluminescence VPCIReader. The readings of each of the three individual experiments are shown by dots. The sample marked “Neg” contains non-circularized DNA with a sequence matching the test DNA circles. To compensate for slide-to-slide variations, the chemiluminescence REEAD signals were normalized to the “Neg” sample and plotted as mean +/− SD. **** = *p* < 0.0001, ordinary one-way ANOVA. “E” refers to an empty well only containing 5′-Amine REEAD primer.

The detection of chemiluminescence REEAD signals shown above was performed using a commercial CCD camera. This option is expensive and not suited for most test laboratories in malaria-endemic areas. Therefore, to enable REEAD detection of malaria in low-resource areas, we designed and constructed a miniaturized, cost-efficient, and portable chemiluminescence assay reader (termed VPCIReader) that provides high-precision image acquisition and analysis capabilities, specifically designed for chemiluminescence REEAD (see Figure 4 and Supplementary Materials S2 for specifications and construction details). The reader was constructed with an integrated battery with an autonomy of approximately 6–8 h and designed to be easily operated through a web application.

The software architecture was developed with four modules: i. the Image Analysis module that interacts with the sensor for image acquisition to finally quantify the REEAD signal, ii. the Data Storage module, iii. the Web Application module that serves as the user interface and links image acquisition and analysis with data storage, iv. the System Configuration.

The performance of the portable chemiluminescence VPCIReader was compared to a commercial CCD camera (Amersham Imager 600) concerning quantitative and sensitive detection of REEAD signals. For this purpose, we used a serial dilution of test circles split in two aliquots each subjected to RCA on two different test slides using the standard chemiluminescence readout protocol as outlined in Figure 1. ECL was applied to each of the slides before they were visualized using the commercial CCD camera (Figure 4B) and the portable VPCIReader (Figure 4C), respectively.

As demonstrated by the graphical depiction of the results of three repetitive experiments (Figure 4B,C, lower panel), the commercial CCD camera and the portable reader gave comparable results when comparing quantifiability and detection limit with the utilized dilutions (note that only the relative readings and not the absolute numbers provided by the two systems can be compared). Concerning the signal-to-noise ratio, a tendency of the portable reader to perform slightly better than the CCD camera was indeed observed. The same tendency was observed when directly comparing the images generated by the two reader systems, where the portable reader produced considerably sharper pictures than the CCD camera (see representative images Figure 4B,C, top panel). Additionally, the VPCIReader web application provides direct access to the intensity values of each rectangular area, while the CCD camera only captures images that necessitate further processing through software-based densitometric analysis. Based on these results, we proceeded to investigate the performance of the portable VPCIReader for the detection of malaria by chemiluminescence REEAD in sub-Saharan Africa.

### 3.4. Detection of Malaria in Saliva from Infected Individuals in Sub-Saharan Africa

**Figure 5:**
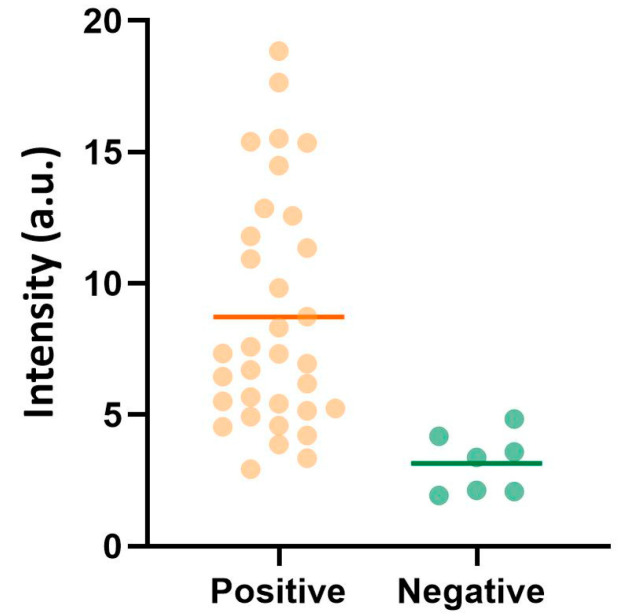
Detection of *Plasmodium* in clinical saliva samples by using VPCIReader. The results obtained by analyzing 33 saliva samples from confirmed malaria-positive individuals and 7 saliva samples from presumed malaria negatives using chemiluminescence REEAD. The average of the results is shown by horizontal lines. Statistics are shown in Supplementary Materials S1B. To compensate for slide-to-slide variations, the chemiluminescence REEAD signals were normalized to a well only containing 5′-Amine REEAD primer.

To investigate the feasibility of chemiluminescence REEAD in combination with a readout using the above-described custom-made portable reader in sub-Saharan Africa, we tested saliva from 33 malaria-positive individuals (confirmed positive with RDT in matching blood) and seven asymptomatic presumed negative individuals at the CERMEL test site, Lambaréné in Gabon [40]. The results shown in Figure 5 demonstrate that most of the saliva samples collected from malaria-positive individuals resulted in signals above signals obtained from presumed negative individuals, and the two groups were found to be significantly different (*p* < 0.0001) using a Kruskal–Wallis test (Supplementary Materials S1B). As evident from the plot in Figure 5 and the table showing the exact readings (Supplementary Materials S1B), four of the samples from presumed negative individuals and approximately seven of the samples of confirmed individuals resulted in readings within the same interval. The relatively low readings in some of the samples from confirmed positive individuals may correlate with lower parasitemia in these individuals. However, as the malaria status of most of the symptomatic individuals was confirmed only by RDT in matching blood, the parasite number is not known. Incomplete extraction of active pTOP1 or suboptimal conditions during transport of some of the samples from rural areas may also explain the relatively low readings. Moreover, the relatively high readings in some of the asymptomatic presumed negative individuals may be the result of these individuals having low *Plasmodium* infection numbers, undetected by the RTD. This is often the case for asymptomatic individuals in populations living in malaria-endemic areas [45].

## 4. Conclusions

In the present study, we present a modified protocol for pTOP1-specific REEAD based on a chemiluminescence readout that allows for the testing of malaria in saliva from suspected individuals in moderately equipped test laboratories. Moreover, we describe the construction and validation of a cost-effective portable reader custom-made for measuring *Plasmodium*-specific chemiluminescence signals obtained by the modified REEAD protocol in malaria-endemic areas. A direct comparison of readings obtained from the same samples using a commercially available CCD camera and the custom-made reader demonstrated that the performance of the developed reader was comparable to the CCD camera for image acquisition and quantification of the REEAD results. Consistently, the portable reader, in combination with the simplified REEAD protocol, was successfully employed to detect the presence of *Plasmodium* parasites in saliva from confirmed malaria-positive individuals in sub-Saharan Africa. Hence, the chemiluminescence REEAD in combination with the availability of an affordable, simple, and portable reader presents a potential new attractive method for diagnosis or screening of malaria using non-invasive samples.

Readings from some of the samples from presumed negative individuals were in the same range as some of the lower readings obtained from samples from confirmed positives. The reason for this overlap is not clear. It could be due to low-level infections in the persons presumed negative in the current study; it could be that some of the samples from positive individuals were damaged during transportation, or that the active enzyme was not extracted efficiently. To address these potential issues, future studies would benefit from improved logistics, such as the use of portable refrigeration or temperature-controlled transport, to ensure the stability of the samples. Additionally, refining enzyme extraction protocols with increased sample size will help reduce potential variability in results.

This study was conducted as a pilot investigation; more comprehensive field trials will be necessary to validate the diagnostic accuracy of the method, assessing its sensitivity and specificity. Future studies should aim to determine the robustness of this system across diverse malaria-endemic settings, investigate its performance in larger, more heterogeneous populations, and optimize the protocol for broader application. With these advancements, the chemiluminescence REEAD method, coupled with the portable reader, could become a valuable tool for malaria screening using non-invasive sample specimens, in resource-limited environments.

## Data Availability

Dataset included in the Supplementary Materials.

## Supplementary Materials

The following supporting information can be downloaded at https://www.mdpi.com/article/10.3390/s24247947/s1: Material S1A: Statistical analysis of data shown in Figure 3B; Material S1B: Statistical analysis of data shown in Figure 5; Material S2: Description and web application User Manual of the portable reader for chemiluminescence REEAD.
