# Supplementary material for: Non-Invasive Malaria Detection in Sub-Saharan Africa Using a DNA-Based Sensor System
Source: Sensors (Basel). 2024 Dec 12;24(24):7947. doi: 10.3390/s24247947 (PMC11680035; doi:10.3390/s24247947)
Supplement: Supplementary file 1 [file sensors-24-07947-s001.zip › sensors-3324130-supplementary.pdf]

Supplementary material S1A

Statistical analysis of data shown in Figure 3B

Statistical analysis of the results shown in Figure 3B.

Negative: negative saliva samples collected in Denmark (No travelling the past two month. Asymptomatic, assumed negative)

Positive: positive saliva samples collected in Gabon (Symptomatic, confirmed by RDT and smear microscopy)

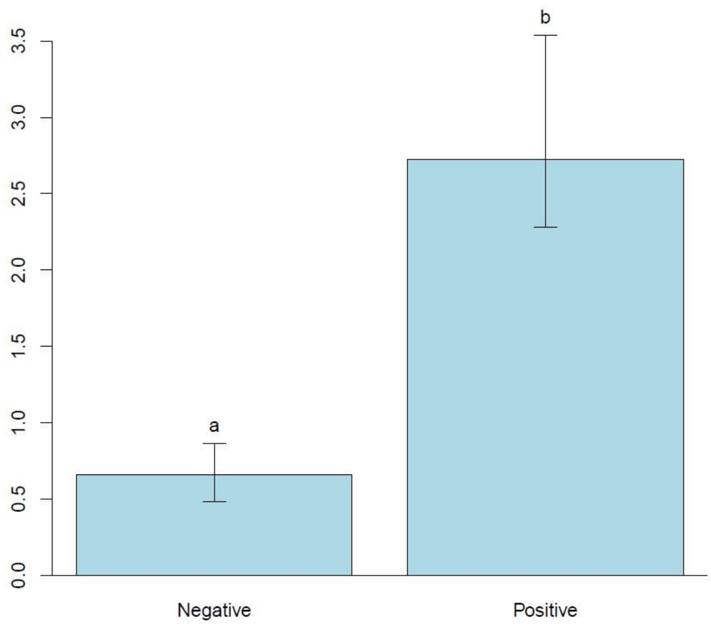

p-value for equality of median = 1.558e-08 (Kruskal-Wallis test)

Levels	Parameter	(95% bootstrap CI)
Negative	0.6584 a	(0.4847-0.8666)
Positive	2.7223 b	(2.2794-3.5399)

Supplementary S1A. Bar chart shows the chemiluminescence REEAD expected values with the corresponding confidence interval (95% coverage) for each sample type. Expected values that are not significantly different at a 0.05 confidence level are marked with the same letter ("a" or "b"). The p-values for pairwise comparisons of the sample types are reported in the lower panel.

Status of samples collected in Gabon and Denmark:

Sample #	Chemiluminiscence REEA signal*	RDT status	Malaria symptoms
1	0,65942	+	+

2	2,53780736	+	+
3	1,70916147	+	+
4	1,91342905	+	+
5	2,34989583	+	+
6	2,47336927	+	+
7	1,68732388	+	+
8	1,29705271	+	+
9	1,64220042	+	+
10	1,89986113	+	+
11	3,28334964	+	+
12	3,79643367	+	+
13	8,47864763	+	+
14	6,82141286	+	+
15	2,32561967	+	+
16	3,24870641	+	+
17	1,43402058	+	+
18	6,10865952	+	+
19	1,60971886	+	+
20	3,8712243	+	+
21	5,53270415	+	+
22	2,90242702	+	+
23	3,22188131	+	+
24	2,54221031	+	+
25	3,95035053	+	+
26	6,90536537	+	+
27	3,22509893	+	+
28	10,2738984	+	+
29	2,23317673	+	+
30	5,5723972	+	+
31	6,227983	N/A	-
32	1,588646	N/A	-
33	0,742204	N/A	-
34	1,109127	N/A	-
35	0,634114	N/A	-
36	1,431901	N/A	-
37	1,281105	N/A	-
38	2,796769	N/A	-
39	0,307219	N/A	-
40	0,866632	N/A	-
41	0,575953	N/A	-
42	0,158326	N/A	-
43	1,609132	N/A	-
44	0,484744	N/A	-
45	0,319875	N/A	-
46	0,257577	N/A	-
47	0,447723	N/A	-

48	0,406055	N/A	-
49	0,388008	N/A	-
50	0,243769	N/A	-
51	0,65835	N/A	-
52	0,940776	N/A	-
53	0,349051	N/A	-
54	0,336395	N/A	-
55	0,579094	N/A	-
56	0,731301	N/A	-
57	2,671865	N/A	-
58	0,909023	N/A	-
59	0,78525	N/A	-
60	0,673623	N/A	-
61	0,48841	N/A	-

Schematic representation of the data obtained from analysis of the saliva samples collected in Gabon and Denmark. *To compensate for slide-to-slice variations, the chemiluminescence REEAD signals were normalized to the average of the negative samples. RDT analysis was performed on blood samples following the manufacturer's manual. Only individuals with malaria symptoms were tested by RDT.

Supplementary material S1B

Statistical analysis of data shown in Figure 5

Statistical analysis of the results shown in Figure 5.

G-Negative: negative saliva samples collected in Gabon (Asymptomatic, assumed negative)

G-positive: positive saliva samples collected in Gabon (Symptomatic, confirmed by RDT and smear microscopy)

The chemiluminiscence REEAD signals were modelled using a Gamma generalised linear model with an identity link function and a classification explanatory variable indicating the sample type (negative from Gabon or positive from Gabon). The adequacy of the model was verified by testing the adherence of the responses transformed by the cumulative distribution function to the uniform distribution yielding a p-value of 0.9305, indicating that there is no significant evidence of lack of model fit. The statistical analysis was performed using the software R version 4.3.2.

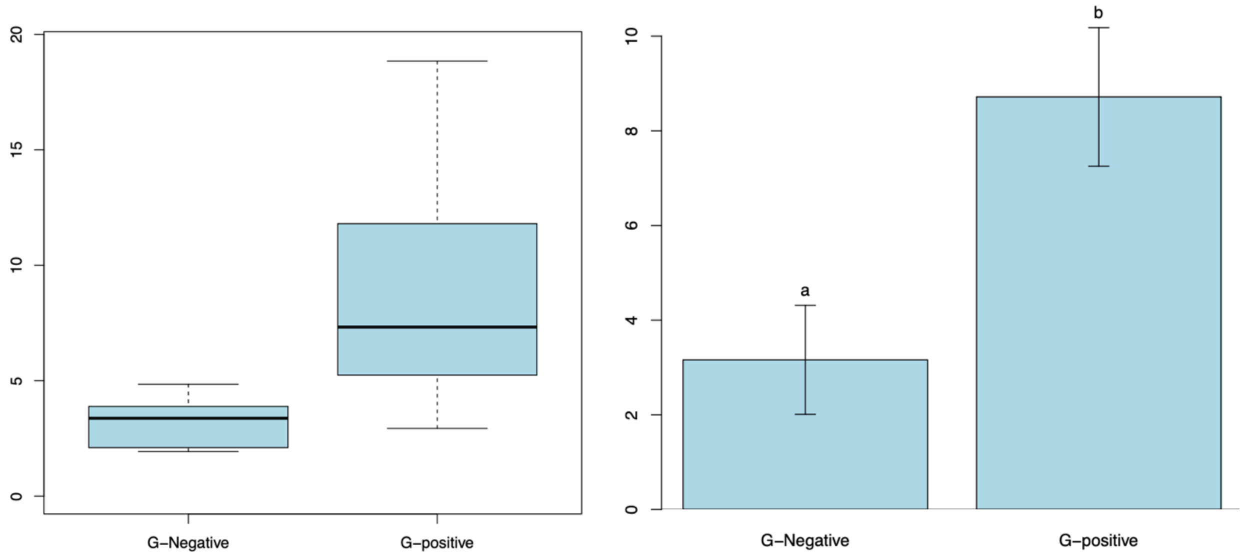

Levels	ParameterCI	
G-Negative	3.16(2.01- 4.31)a	
G-positive	8.72(7.25-10.18)b	p < 0.00001

Supplementary S1B. Left panel, shows a boxplot of the chemiluminescence REEAD expected values with the corresponding confidence interval (95% coverage) for each sample type (the median is indicated by a black line). Right panel. Bar chart depiction of the same results. Expected values that are not significantly different at a 0.05 confidence level are marked with the same letter (“a” or “b”). The p-values for pairwise comparisons of the sample types are reported in the lower panel.

Status of samples collected in Gabon:

Sample #	Chemiluminiscence REEA signal*	RDT status	Malaria symptoms
1	15,39747	+	+
2	14,48962	+	+
3	7,321109	+	+
4	9,825459	+	+
5	5,671939	+	+
6	6,18578	+	+
7	3,356034	+	+
8	6,944012	+	+
9	15,51216	+	+
10	12,57853	+	+
11	8,739034	+	+
12	10,93636	+	+
13	8,322141	+	+
14	7,330218	+	+
15	17,65116	+	+
16	12,85789	+	+
17	15,35442	+	+
18	11,35302	+	+
19	11,80149	+	+
20	18,84245	+	+
21	7,595950	+	+
22	6,706603	+	+
23	5,240724	+	+
24	4,216865	+	+
25	5,150018	+	+
26	6,452939	+	+
27	4,544069	+	+
28	2,934192	+	+
29	4,935651	+	+
30	3,879539	+	+
31	4,595305	+	+
32	5,508909	+	+
33	5,431509	+	+
34	3,373555	N/A	-
35	1,930849	N/A	-
36	2,080880	N/A	-
37	4,845736	N/A	-
38	4,184930	N/A	-
39	3,588835	N/A	-
40	2,123777	N/A	-

Schematic representation of the data obtained from analysis of the saliva samples collected in Gabon. *To compensate for slide-to-slice variations, the chemiluminescence REEAD signals were normalized to a well only containing primer. RDT analysis was performed on blood samples following the manufacturer’s manual. Only individuals with malaria symptoms were tested by RDT.

Supplementary material S2

Description and Web application User Manual of the portable reader for chemiluminescence REEAD.

Design and Construction of the Reader

The VPCIReader is a miniaturized and portable chemiluminescence assay reader that provides high precision image acquisition and analysis capabilities, specifically designed for VPCIR chemiluminescence assays.

VPCIReader has an integrated battery, with an autonomy of approx. 6-8h and a charge time of 1h, and a power supply.

VPCIReader is easily operated through a web application. This web app runs on the VPCIReader itself, and is only accessible through a Wireless LAN, for which the VPCIReader acts as an access point. VPCIReaders can be securely accessed remotely, when connected to a network with internet access.

Regarding data storage, everything (web app data, raw images, VPCIReader software) is in a USB drive inside the VPCIReader.

The software architecture was developed with 4 modules: i. the Image Analysis module that interacts with the sensor for image acquisition to finally quantify the plates signal (all its functionality is exposed through a simple REST API), ii. the Data Storage module for sensor data and for human-generated one (experiments, types of plates, samples), iii. The Web Application module that serves as the user interface and links image acquisition and analysis with data storage, iv. the System Configuration: OS-level packages, networking services, the components above.

Image analysis overview

Each experiment produces the following types of images:

- Dark Frames: obtained with the tray closed just before measuring the plate.
- Raw Frames: images taken with the plate inside
- Corrected Frames: the result of correcting raw frames using the dark ones
- Display Frames: synthetic images of the corrected frames, meant for naked eye validation and grid placement

The sensor data for each well corresponds thus to a rectangular region of the original frame.

Every well is quantified independently, using as input blue channel readouts of its corresponding region in each corrected frame. As the sensor response is linear, the number of readouts is the same for each well, and dark frame correction removes virtually all background distortion and constant noise, we can simply add all the readouts of each region.

For image acquisition we always work with the RAW output generated in DNG format by the camera. The DNG files are then converted to 16-bit TIFF files, so that the output corresponds at each pixel with the sensor readouts, without any modification. After this, the raw TIFF files are cropped to a region fitting the whole tray, rotated so that the orientation matches that of the user looking at the VPCIReader, and only the blue channel is extracted.

For wells, we work with a rectangle of approx. 5000 pixels. As they are sized (X3mm, Y1.5mm) we are observing around 103 pixels per mm^2.

VPCIReader

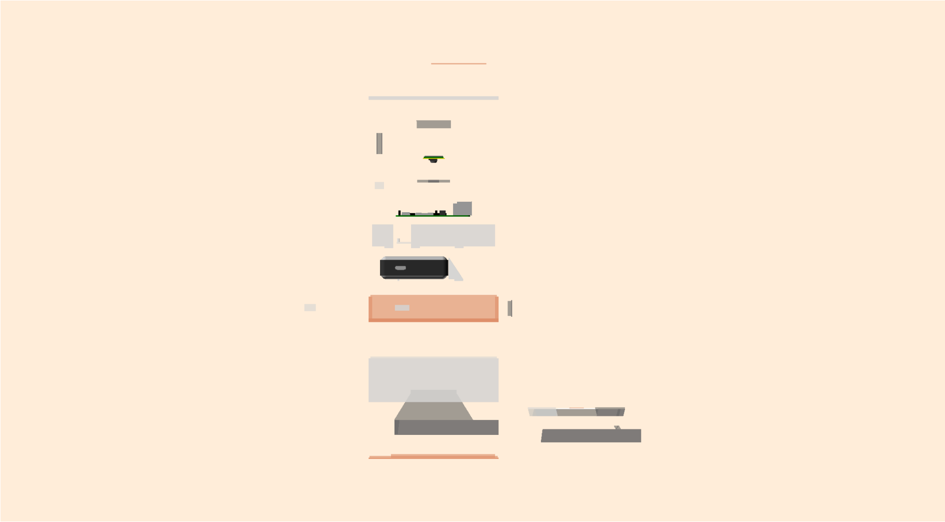

Technical Documentation – v0.1.1

October 5, 2023

Eduardo Pareja-Tobes

NISOLAB

Contents

1 Summary 1
1.1 VPCIReader Operation 2
1.2 Remote Access 3

2 Software 4
2.1 Architecture 4
2.1.1 Components 4
2.2 System Configuration 4
2.2.1 Custom OS Image 4
2.2.2 Propellor 5
2.3 Image Analysis 6
2.3.1 Overview 6
2.3.2 Image Acquisition 7
2.3.3 Image Analysis 9
2.3.4 API 11
2.4 Web Application 12

3 Hardware 13
3.1 Electronic Components 13
3.2 3D Printed Parts 16
3.2.1 Electronics Box 18
3.2.2 Dark Box 21
3.2.3 Plate Tools 21
3.2.4 Parts List 24

A Electronics Setup 25
A.1 Battery 25
A.2 Raspberry Pi 26
A.3 Camera 27
A.4 Fan 27

1 Summary

> *This document is **not** written with **users** in mind, and assumes familiarity with what the VPCIReader is, its use, and technical issues around it. Users should probably be referred to the Web Application User Manual instead.*

The VPCIReader is a chemiluminescence assay reader prototype. It provides best-in-class image acquisition and analysis capabilities, specifically designed for VPCIR chemiluminescence assays.

VPCIReaders are operated through a web application: using any WiFi-capable device[1], the user connects to a VPCIReader-specific WiFi network[2] and then everyhting about working with the VPCIReader –managing users, registering samples, reading plates... can be performed through a web interface, available at `http://reader<n>.local`.

VPICReaders have an integrated battery, with an autonomy of approx 6-8h and a charge time of 1h, and a power supply. Thanks to the design and the specific battery used, the behavior is equivalent to that of a tablet or a laptop from a user perspective: you can plug or unplug the power supply at any time, and the VPCIReader will keep working for as long as the battery has enough charge.

Regarding data storage, everything (web app data, raw images, VPCIReader software) is in a USB drive inside the VPCIReader. This USB drive is easily replaceable in case of failure, and can be shipped for recovery or offline update.

[1] Essentially any laptop, desktop, smartphone, or tablet will do.
[2] Note that no internet access of any kind is required.

1.1 VPCIReader Operation

The VPCIReader is operated through a web application. This web app runs on the VPCIReader itself, and is only accessible through a Wireless LAN[3] , for which the VPCIReader acts as an access point.

For normal operation thus the user connects to the specific WLAN of the VP-CIReader which he wants to operate[4], and opens `http://reader{n}.local` in a browser.

WEB APP ACCESS

The access data for the VPCIReader WLAN is

- **SSID** `vpcireader-{n}`
- **password** `hola reader {n}`

So the WiFi SSID is `vpcireader-001` , and the password is `hola reader 001` , for $n = 1$.

This WiFi network is a $2.4 GHz$ `802.11g` network, using channel 8. Commonly $2.4 GHz$ WiFi networks use channels 1, 6, and 11; the choice of channel 8 reduces the likelihood of WiFi signal congestion in a generic crowded environment.

The VPCIReader can thus be operated by virtually any device with a browser[5], and capable of connecting to a `802.11.g` network.

If the client supports mDNS[6], the VPCIReader web app is accessible within the VPCIReader WiFi at

- `http://reader{n}.local`

---

[3]It should be remarked that this WLAN has no internet access, and it is only meant to be used for operating the corresponding VPCIReader.

[4]VPCIReaders are identified with a number in the $[1, 999]$ range, written with three digits: `001, 007, 032, 124` etc. This number appears in a black tab on the front top right corner.

[5]The web app itself is written in raw JavaScript, and does not require any advanced features: any browser with JavaScript suppport will do.

[6]mDNS is likely to be supported in any device other than possibly Windows and old Android phones.

If mDNS is not enabled or supported by the OS, the user can still access the web app at

- `http://10.0.0.5`

Several readers can be operated at the same physical location, only limited by WiFi signal interference.

1.2 Remote Access

VPCIReaders can be securely accessed remotely, when connected to a network with internet access.

Remote Access can be used for

- **Software Update** Both the image analysis system and the web app can be remotely updated securely as soon as the VPCIReader has internet access.
- **Remote Operation** The web app can be accessed through remote port forwarding, making it possible to operate a VPCIReader remotely. It can thus be used to provide support, diagnostics, etc.
- **Data Backup and Recovery** Apart from the export functionality of the web app, all data can be exported remotely, or recovered in case of a malfunctioning device.

REMOTE ACCESS IMPLEMENTATION

The VPCIReader will try to connect (every minute) to a WiFi connection with

- **SSID** `4g-vpcir-reader`
- **password** `charm-rotten-jumble-other`

Once connected to this network, its own VPCIReader WiFi will be shut down, and a persistent reverse SSH tunnel to a bastion host will be established. Administrators in possession of the corresponding secret keys can then log in through this bastion host.

It is also possible to use a ethernet connection; the cable can be connected by removing the tab with the number in front.

2 Software

All software runs on the Raspberry Pi board: image analysis, web application, and data storage.

2.1 Architecture

2.1.1 Components

We will distinguish the following modules

- **Image Analysis** This component interacts with the sensor for image acquisition, works with the sensor data, and ultimately quantifies plates. All its functionality is exposed through a simple REST API.
- **Data Storage** For both sensor data and human-generated one: experiments, types of plates, samples, etc.
- **Web Application** Serves as the only user interface, and links image acquisition and analysis with data storage.
- **System Configuration** OS-level packages, networking services, the components above –all this must be installed and configured on the Raspberry Pi board.

2.2 System Configuration

2.2.1 Custom OS Image

The system is configured starting from a custom 64-bit Raspberry Pi OS Lite image. Significant changes with respect to the standard image are

- key-based SSH `root` login enabled
- A 4GB swap file configured

2.2.2 Propellor

Starting from that image, the system is configured using propellor, a configuration management system based on Haskell and Git. The key advantages for us are

- **Git-backed Configuration** The whole system configuration is stored in a git repository, which is furthermore part of the system itself.
- **Complex Configuration Support** Generating systemd services, complex networking scripts for the VPCIReader network setup –all of it is version-controlled and generated in Haskell.
- **Integrated Components** The whole database schema is specified as code using a homegrown Haskell DSL, and then created by generating SQL scripts using again Haskell.
- **Security** We have the advantages of an immutable OS image while conserving programmability; and for example private keys can be encrypted and version-controlled within propellor, using a master key in an external secure device[1].

PROPELLOR CONFIGURATION

All the VPCIReader system configuration: software, components, network services, the autohostspot system, database tables and configuration, … are specified using propellor together with some custom extensions. As a sample, this is the top-level function returning the configuration for a VPCIReader numbered `n` with public key `pbk` .

```
readerProps n pbk = props
    & Hostname.sane
    & osDebian (Stable "bullseye") X86_64
    & Apt.installed
      [ "g++"
      , "gcc"
      , "libc6-dev"
      , "libffi-dev"
      , "libgmp-dev"
      , "make"
      , "xz-utils"
```

[1]We are using a Nitrokey for this, for example.

```
      , "zlib1g-dev"
      , "git"
      , "gnupg"
      , "netbase"
      , "libnuma-dev"
      , "dcraw"
      ]
    & Ssh.userKeys root hostContext [(Ssh.SshEd25519, pbk :: String)]
    & ABN.manualInterface "wlan0" -- managed by autohotspot below
    & dhcp "eth0"
    & User.accountFor vpcireaderUser
    & Postgres.installed
    & Postgres.hasUser vpcireaderUser
    & Postgres.hasDatabase db
    & Postgres.createDatabaseObjects db
    & hasInitialData
    & Git.repoConfigured home ("receive.denyCurrentBranch", "updateInstead")
    & postgRESTConfFile `File.hasContent` lines postgRESTConf
    & serviceEnabledAndRunning postgRESTService postgRESTServiceDef
    & serviceEnabledAndRunning vpcirService vpcirServiceDef
    & AutoHotspot.withAutoHotspot
        [ ("4g-vpcir-reader","charm-rotten-jumble-other") ]
        ([i|vpcireader-00#{n :: Int}|] :: String, [i|hola reader 00#{n :: Int}|] :: String)
```

FIRST TIME SETUP

Once the Raspberry Pi board with the initial image is running and network accessible, we just need to (in propellor parlance) spin the corresponding host

```
# reader${i}.vpcireader should resolve to the pi we want to configure
# use etc/hosts or any other mechanism
propellor --spin reader${i}.vpcireader.com
```

Propellor itself will be bootstrapped, Haskell and the relevant dependencies installed, and our configuration applied. This first time setup will take several hours –the Raspberry Pi processor is fairly limited.

2.3 Image Analysis

2.3.1 Overview

Each experiment produces the following types of images

- *Dark Frames* Obtained with the tray closed just before measuring the plate.
- *Raw Frames* images taken with the plate inside
- *Corrected Frames* the result of correcting raw frames using the dark ones
- *Display Frames* Synthetic images of the corrected frames, meant for naked eye validation and grid placement

The sensor data for each well corresponds thus to a rectangular region of the original frame.

As everything regarding the plates (assembly, locking, tray placement, etc) is a manual process, small variations in position are to be expected. This is not an issue though, as

1. The rectangular regions are big enough to accommodate a slightly displaced well
2. The user can adjust the whole grid for each experiment through the web application, moving it at steps of $\approx 0.1mm$ in every direction.

Every well is quantified independently, using as input blue channel readouts of its corresponding region in each corrected frame. As the sensor response is linear, the number of readouts is the same for each well, and dark frame correction removes virtually all background distortion and constant noise[2], we can simply add all the readouts of each region.

2.3.2 Image Acquisition

Images are acquired using the Raspberry Pi `libcamera` -based suite of camera applications. We explicitly set all options to values that will guarantee no software-level image processing takes place, the image is acquired as fast as possible, and generate a full camera settings json metadata file. We always work with the RAW output generated in DNG format by `libcamera-still` . These DNG files are then converted to 16-bit TIFF files using `dcraw` , so that the output corresponds at each pixel with the sensor readouts, without any modification. As an extra safety measure, we disable all Raspberry Pi board LEDs before taking any image.

[2]hot and stuck pixels are the most worrying.

After this, the raw TIFF files are cropped to a region fitting the whole tray, rotated so that the orientation matches that of the user looking at the VPCIReader, and only the blue channel is extracted.

libcamera-still FRAME ACQUISITION

```
#  --verbose        Set verbosity level. Level 0 is no output, 1 is default, 2 is verbose.
#  --metadata arg   Save captured image metadata to a file or "-" for stdout
#  --nopreview      Do not show a preview window
#  --sharpness arg  Adjust the sharpness of the output image, where 1.0 = normal sharpening
#  --contrast arg   Adjust the contrast of the output image, where 1.0 = normal contrast
#  --ev arg         Set the EV exposure compensation, where 0 = no change
#  --brightness arg Adjust the brightness of the output images, in the range -1.0 to 1.0
#  --saturation arg Adjust the colour saturation of the output, where 1.0 = normal and
#                   0.0 = greyscale
# --denoise arg     Sets the Denoise operating mode: auto, off, cdn_off, cdn_fast, cdn_hq
# --awbgains arg    Set explict red and blue gains (disable the automatic AWB algorithm)
# --raw             Also save raw file in DNG format
# --encoding arg    Set the desired output encoding, either jpg, png, rgb, bmp or yuv420
libcamera-still --verbose --metadata "${i}.json"      \
  --immediate --nopreview                             \
  --sharpness 0 --contrast 1.0 --ev 0 --brightness 0  \
  --saturation 0 --denoise off --awbgains 1.0,2.0     \
  --raw --encoding png --output "${i}.png"            \
```

DNG TO TIFF CONVERSION

We use dcraw extracting the raw sensor data as a grayscale image, with no interpolation or scaling and a linear (1, 1) gamma correction curve. The full command together with the details for each option is shown below

```
#   -v    Print verbose messages, not just warnings and errors.
#   -d    Show the raw data as a grayscale image with no interpolation.
#         Good for photographing black-and-white documents.
#   -D    Same as -d, but with the original unscaled pixel values.
#   -4    Linear 16-bit, same as -6 -W -g 1 1.
#   -T    Write TIFF with metadata instead of PGM/PPM/PAM.
#   -j    For Fuji Super CCD cameras, show the image tilted 45 degrees.
#         For cameras with non-square pixels, do not stretch the image
#         to its correct aspect ratio. In any case, this option guarantees
#         that each output pixel corresponds to one raw pixel.
dcraw -v -D -4 -j -T frame.dng
```

Final tray area extraction

With the sensor data 16-bit TIFF as input, we

1. *Extract tray area* a rectangle with top left corner `(320,240)` and height and width `(2640,1984)`.
2. *Adjust orientation* this tray region is then rotated 270 degrees so that its orientation matches that of the user looking at the front of the VPCIReader.
3. *Extract blue channel* As the luminol reaction emits light at around $425nm$, we extract the blue channel following the RGGB Bayer pattern of the Sony IMX219 sensor.

This, as the rest of the image analysis module, is implemented in Haskell. The camera in use, resolution, Bayer pattern etc are part of the module configuration, and could be easily modified to support different sensors by just specifying the relevant parameters.

For wells, we work with a rectangle of approx. 5000 pixels. As they are sized ($X3mm$, $Y1.5mm$) we are observing around 10^3 pixels per mm^2. Put another way, each pixel covers on the order of a square of $30\mu m$ side[3].

2.3.3 Image Analysis

All the image analysis proper has as input the raw frames we have just described.

Dark frame correction

We subtract the single dark frame from each of the ten raw frames; this subtraction is of course truncated.

We have found experimentally that transient effects such as hot pixels are significant, while differences in sensitivity across pixels are fairly small, and on the

[3]These back of the envelope calculations correspond to what one pixel covers, but it should be taken into account that we can only use the blue channel; we do not have image data from the area corresponding to the other channels.

whole have a small effect: each well makes use of thousands of pixels. Furthermore, variations intrinsic to the chemiluminescence reaction dwarf any difference in pixel sensitivity.

We have thus dispensed with the implemented flat field correction, significantly simplifying the setup process, while keeping dark frame correction. It should also be noted that as the dark frame is obtained just before the raw frame acquisition and is thus experiment-specific, it will also correct (at least up to a point) dynamic effects due to, for example, temperature variations.

Display frames

The purpose of display frames is twofold: first, they serve as naked eye validation, letting the user easily estimate the relative intensities between wells, or the presence or absence of signal thereof. Secondly, by superposing on top a grid with the well regions, the user can match these regions with the wells signal, having a visual guarantee that any further analysis actually corresponds to the signal in each well.

For this, each (corrected) frame is normalized to the maximum pixel value across the set of corrected frames[4], increasing contrast for plates with measurable but low absolute signal.

Well intensity quantification

Given a grid position, each well is quantified for each of the 10 corrected frames, by extracting the corresponding region and adding all the pixel readouts in that region.

Data layout

Every camera frames folder contains

[4]using dark frame corrected frames here is key, as otherwise this normalization won't be effective due to the almost certain presence of hot or stuck pixels with close to saturated readouts.

1. `takeframes.sh` The script that was used for obtaining the frames through `libcamera-still` and posterior conversion from DNG to TIFF with `dcraw` , with the aforementioned settings.
2. `${i}.dng` the DNG files, one per frame
3. `{i}.json` `libcamera` image acquisition metadata, including timestamps, exposure, gain, etc.
4. `${i}.tiff` the file containing the pure raw sensor data.

2.3.4 API

Interaction with the image analysis module is done through a simple REST API, with the following endpoints and methods:

```
GET plate/${pt}/grid-image
```

Returns the grid of well rectangles corresponding to the provided plate type `pt` , as a transparent png image. Returns `Content-Type: image/png` , `plate_type` in the route must be one of `C4_R5` , `C4_R10` .

```
POST experiments/${ex}/dark-frames
```

Acquires the dark frames for the given experiment `${ex}` , and returns their name and URL as JSON.

```
POST experiments/${ex}/raw-frames
```

Acquires the raw frames for the given experiment `${ex}` , and returns their name and URL as JSON. Will fail if the experiment has no dark frames associated with it.

```
POST experiments/${ex}/display-frames
```

Generates the display frames for the given experiment `${ex}` , and returns their name and URL as JSON. Calling this method twice will generate the same display frames anew. Will fail if the experiment has no raw frames associated with it.

```
POST experiments/${ex}/measure?plate=${pt}&col_disp=x&row_disp=y
```

Quantifies the corresponding plate, treating it as having type `pt`, and placing the grid according to `x` and `y`. Returns a JSON representation of the quantified plate, the result of each well for all raw frames. Will fail if the experiment has no raw frames associated with it.

2.4 Web Application

The web application is implemented in JavaScript, and interacts with both the image analysis component and the postgres database, keeping image and experiment metadata coherent. This interaction is done for image analysis through the API outlined above, while for the database we use postgREST, which let us access the postgres server through a REST API.

At the transport level, postgREST, the image analysis API, and the web application and static files (images, etc), are all served through a custom Warp server. All the code for this, both client-side and server-side, are part of the same repository.

Client-side are minimal: any device with a JavaScript-capable browser should be enough for controlling the VPCIReader through the web application.

For more details we refer to the *VPCIReader Web Application Manual*.

3 Hardware

We consider hardware all the electronic components, externally supplied, but also all the custom built pieces, 3D-printed in-house. The total cost, in terms of supplies, is approximately 250-300€ per VPCIReader.

3.1 Electronic Components

The total cost in electronic components is approx 200-250€. Most of them should be easily sourced; only the Raspberry Pi board, due to the sadly recurrent supply issues, and the Voltaic battery, because of the importing and shipping restrictions for batteries, can be hard to obtain.

RASPBERRY PI 4 MODEL B - 4GB

- **Website** Raspberry Pi 4 Model B.
- **Compliance** CE Mark, FCC approved. Further info at Raspberry Pi 4B - Compliance.
- **Cost** Approx 90€
- **Supplier** Raspberry Pi OEM channels.

RASPBERRY PI CAMERA MODULE V2

- **Website** Raspberry Pi Camera Module 2
- **Compliance** CE Mark, FCC approved. Further info at Raspberry Pi Camera Module 2 - Compliance.
- **Cost** Approx 35€
- **Supplier** Kiwi Electronics.

VOLTAIC V25 BATTERY

- **Website** Voltaic V25.
- **Compliance** CE Mark, FCC approved. Further info at Voltaic V25 - Specifications.
- **Cost** Approx 75€ including import taxes.
- **Supplier** Kiwi Electronics, Funky Leisure.

RASPBERRY PI POWER SUPPLY 5.1V/2.5A MICROUSB

- **Website** Raspberry Pi 1, 2 and 3 Power Supply
- **Compliance** CE Mark
- **Cost** Approx 12€.
- **Supplier** Kiwi Electronics.

SAMSUNG USB FIT PLUS 256GB

- **Website** FIT Plus USB 3.1 Flash Drive 256GB
- **Compliance** CE Mark, FCC Approved.
- **Cost** Approx 40€.
- **Supplier** Any electronics consumer store.

RASPBERRY PI CASE FAN

- **Website** Raspberry Pi 4 Case Fan.
- **Compliance** CE Mark, FCC approved.
- **Cost** Approx 6€.
- **Supplier** Kiwi Electronics.

MICROUSB TO USB-C ADAPTER

- **Website** Raspberry Pi USB Micro-B to USB-C Adapter.
- **Compliance** N/A.
- **Cost** Approx 2€.
- **Supplier** Kiwi Electronics.

OTHER COMPONENTS

- **USB-A to USB-C cable** A short (10–15*cm*) with a flat right-angle USB-A end, for connecting the Raspberry Pi board to the battery.
- **CPU Heatsink** included as part of the case fan.
- **Blackout Tape** Advance Tapes - AT205, a non-reflective, flame-retardant aluminum tape. Available from Thor Labs.
- **Double Sided Tape** Generic permanent tape such as Nitto Denko - Double Sided Adhesive Tape.

3.2 3D Printed Parts

All parts are designed in OpenSCAD and are fully parametric. The global dimensions of the box corresponds to an A5 paper, simplifying shipping and handling. Furthermore, these dimensions are so that everything can be printed as one part in a Prusa i3 MK3S+.

PRINTING

All parts are meant to be printed with PETG filament. The three colors used are

- **White** Prusament PETG Signal White
- **Black** Prusament PETG Matte Black
- **Orange** Prusament PETG Prusa Orange

Functionally it is key that all the parts that make up the dark box are printed using a truly opaque filament, such as the matte black above; most PETG filaments are translucent, as PETG without any additives is transparent.

Colors are chosen based on both functionality and aesthetics considerations, while maintaining a balanced use of each filament.

The printing process is designed and optimized for batches of **four** VPCIReaders; parts can be printed in approx 10 days with a single Prusa i3 MK3S+, consuming one 1*kg* filament roll per color (three in total)[1].

A full set of `PrusaSlicer` projects and `gcode` s for printing one batch has been carefully optimized for a (well-calibrated) Prusa i3 MK3S+ with a stock nozzle and a textured sheet[2], with a focus on dimensional accuracy. All parts print without supports but for `box` , `dark_box_cover` , and `dark_box` , which use them only sparingly. Apart from removing these supports, which can be easily done, no part post-processing is required.

[1]With just a single printer then it is possible to produce around 150 VPCIReaders per year (assembly and configuration can be done in parallel, of course).

[2]Apart from a thorough Z calibration, the use of a filament dryer *while printing* is strongly advised; printing pieces with small features or a complicated geometry using PETG requires moisture levels below 20–25%.

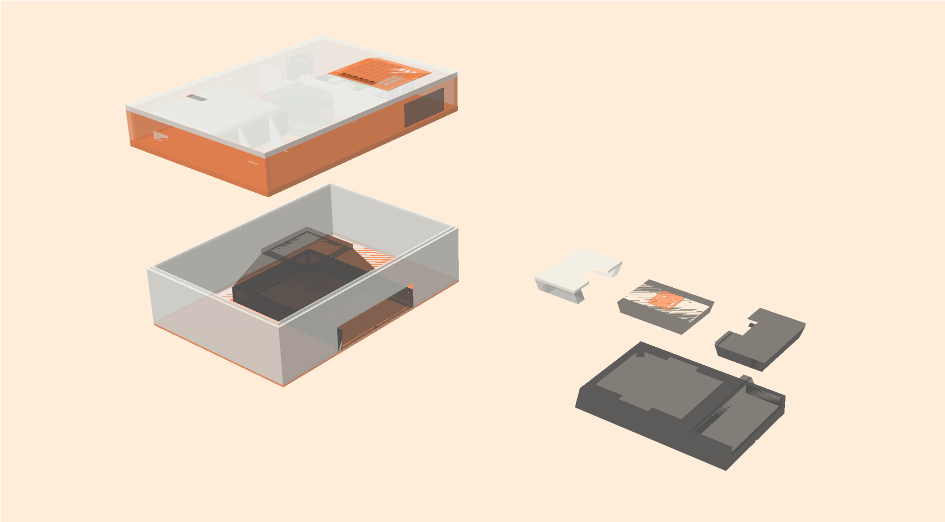

Exploded view of the Electronics Box, the Dark Box, and the tray together with some plate tools.

VPCIREADER STRUCTURE

The VPCIReader can be separated into three modules:

- **Electronics Box** this box holds all the electronic components: camera, board, fan, battery, and necessary cables and adapters. At the top we have ventilation grid, and battery charge indicator. A black tab at the front displays the VPCIReader number, the power button is on the right side, and the microUSB power input is at the back.
- **Dark Box** for housing the tray, establishing a completely dark environment, and setting the plate at an optimal distance from the camera.
- **Plate Tools** The tray, plate assemblers, lockers, and adapters.

The Electronics Box and the Dark Box are press-fitted, and can be manually removed for inspection, shipping, independent repair, or even the use of new future modules instead of a dark box.

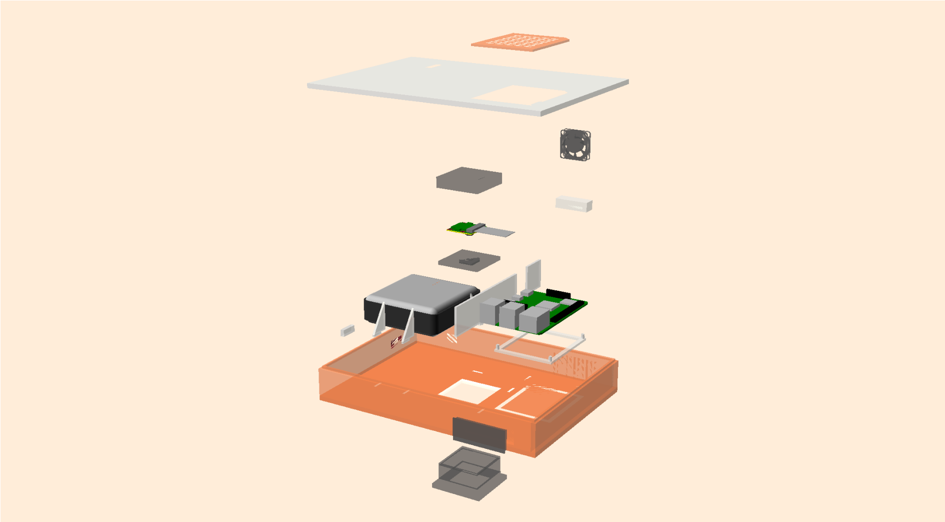

Exploded view of the Electronics Box including parts and electronic components.

3.2.1 Electronics Box

This box houses all the electronic components. The orange `box` has several slots inside, on which holders and tabs for the electronic components are press-fitted[3]. The white `box_top` and the `ventilation_grid` on top are also press-fitted, and can be removed manually with the help of a pry tool.

BATTERY

The battery is held in place on the *XY* plane by three tabs (one `battery_right_tab` and two `battery_back_tab`), while on the *Z* axis the bottom of the `box` and the `box_top` press against it. On the back we have the microUSB to USB-C adapter, fixed in place by the `microUSB_holder`. On the left the `power_button` is glued to the one on the battery[4]. The `box_top` has a hole that makes the integrated battery lights visible from outside.

[3]While all parts can simply be press-fit assembled, some of them should also be glued as a precautionary measure, using the double-sided tape or a generic adhesive.

[4]Pushing this button once displays the battery charge level, while doing it twice will turn the VP-CIReader on/off.

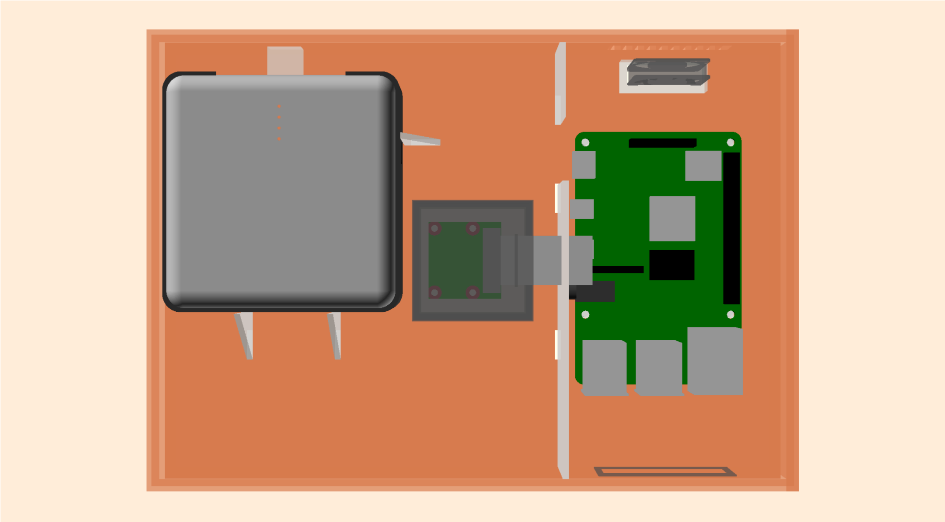

Top view of the assembled electronic components inside the Electronics Box.

RASPBERRY PI BOARD AND FAN

The `pi_board_holder` is press-fitted onto the `box` bottom, and then the Raspberry Pi board fixed to it by fitting the holes to the rods on the corners[5]. The board is placed so that by removing the `front_cover` we can plug an ethernet cable or remove the USB drive.

On the back, the case fan is fitted to the `box` using the `fan_holder` . There is an air intake at the back of it, and the `ventilation_grid` is placed on top of the board microprocessor and ports; the two rectangular white separators on the left (`pi_board_separator_1` , `pi_board_separator_2`) isolate the board region from the rest of the box. All this[6] guarantees good airflow and thus adequate board cooling, while at least isolating partially the camera module from the temperature changes caused by the board.

[5]The board can be secured further by glueing it to the rods, using any cyanoacrylate-based glue –i.e. superglue. In any case, this should be done with the board off, and at a cool temperature. The `pi_board_holder` can also be glued to the `box` slot.

[6]Note that in the assembly process, once the USB-A to USB-C cable is connecting the battery with the board is set, the gap between the two separators will be covered with tape.

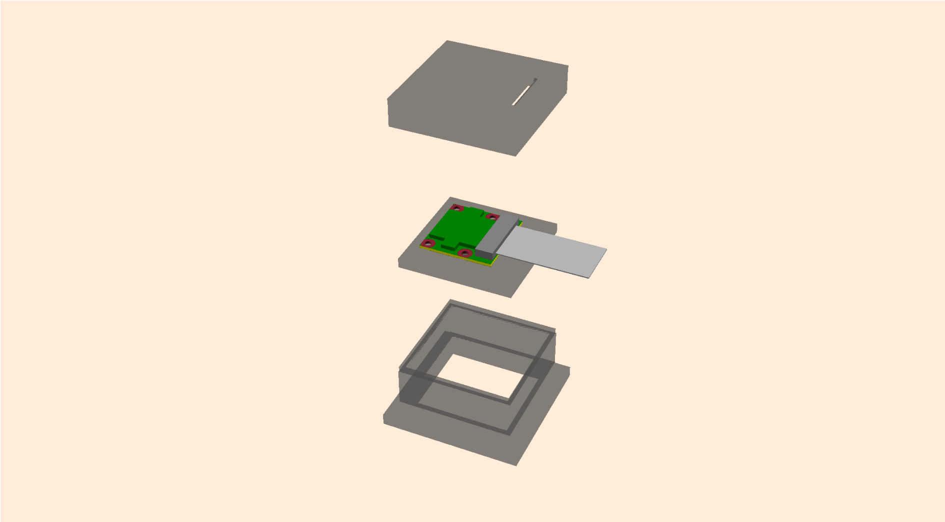

Close-up of the camera already fitted to `camera_box_bottom`*, with* `camera_inner_tube` *below. On top We can see the slit on* `camera_box_top` *.*

CAMERA

The `camera_inner_tube` fits tightly to the `box` , while the camera itself is locked in place by fitting the lens cover inside the `camera_box_bottom` . This needs to be covered by the `camera_box_top` ; a small amount of light leaks through the camera circuit board otherwise. The camera cable goes through a slit at the top of the `camera_box_top` , and then passes between the top of the `pi_board_separator_1` and the `box_top` before, connecting to the board.

Once assembled, both the joint between the `camera_inner_tube` and the `camera_box_top` , and the cable going out of the `camera_box_top` slit must be secured using blackout tape.

FRONT COVER

At the front right side there is a small black `front_cover` , with the VPCIReader number overlaid in white. This cover can be manually removed, giving access to the USB drive and the ethernet port; a standard RJ45 cable can be pass through the exposed hole.

3.2.2 Dark Box

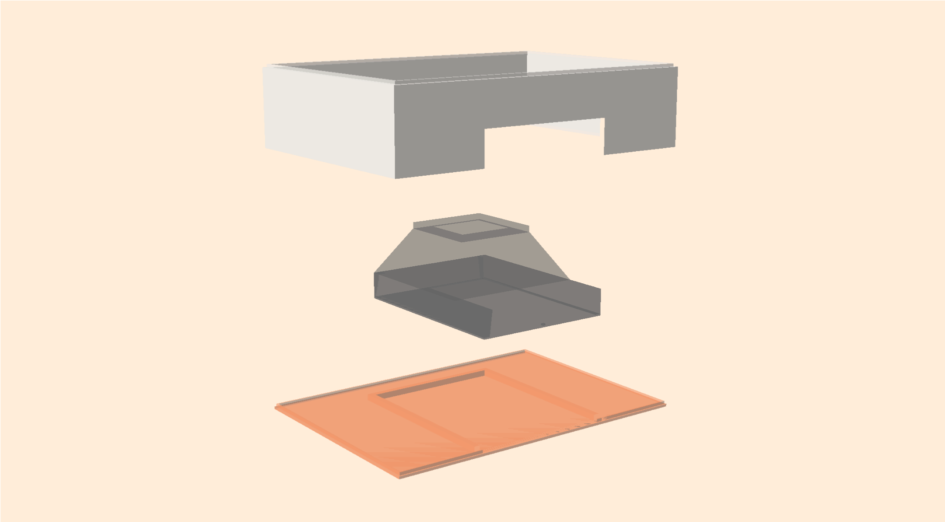

The height of the `dark_box` is derived from the minimum object distance for our sensor[7] together with the size of the plates: we want to maximize resolution without risking placing the plate out of the field of view, or pushing it too close to the edges. The angles of the pyramid-like part are computed in terms of the camera angles of view; this shape saves material, and simplifies 3D printing.

The `dark_box` press-fits with the `camera_inner_tube` (part of the Electronics Box) above, and with the `bottom` part below. The `dark_box_cover`, fitting with the `box` above and the `bottom` below, not only fulfills an aesthetic function; it also provides stability to the whole assembly.

3.2.3 Plate Tools

PLATE ASSEMBLERS

Plate assemblers let the user assemble a plate which can then be locked by the plate adapters in a fixed position, making its reading by the VPCIReader possible.

[7] As the lens is adjusted manually, and can pop out if unscrewed too far, we need to err on the side of caution for this.

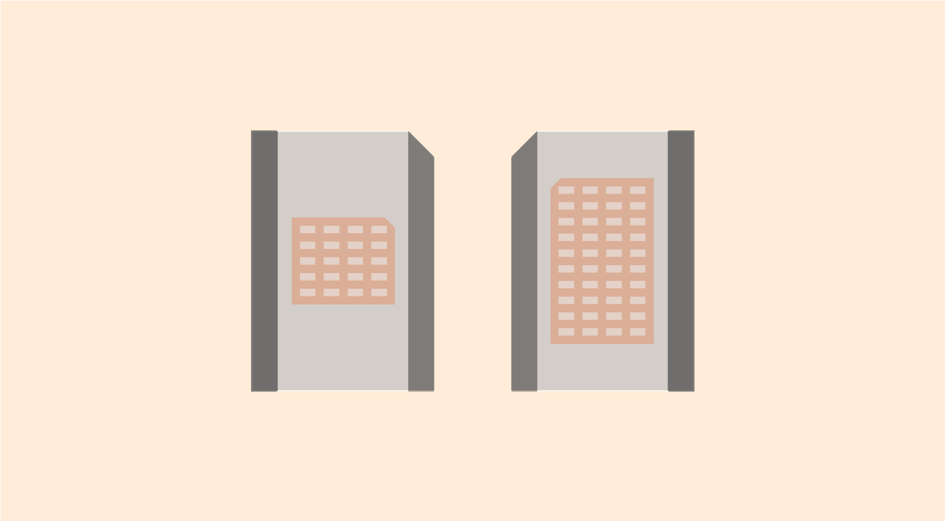

Top view of the two plate assemblers corresponding to each plate type: `plate_assembler_r5_c4` *and* `plate_assembler_r10_c4` *, together with matching assembled plates on top. Note that the two types of plate have opposite orientations.*

The glass slide has standard *X* and *Z* dimensions, but on the *Y* axis neither shape nor dimensions are fixed, as the slide is cut manually by the user. The

Assuming only a maximum glass slide y length of `50 mm` , the plate assemblers let the user glue the silicone to the slide at a fixed x position and a right y angle. The plate adapters will then fix the y position.

There are two plate assemblers, one for each plate type:

- `r5_c4_assembler`
- `r10_c4_assembler`

PLATE ADAPTERS

Each adapter is made of three parts: one holder `plate_holder` on which the plate gets fixed in every direction but the Y axis, and two lockers, `plate_locker_top` and `plate_locker_bottom` , which together fix the Y position by sliding across the holder and fitting them against the orange silicone part of the plate.

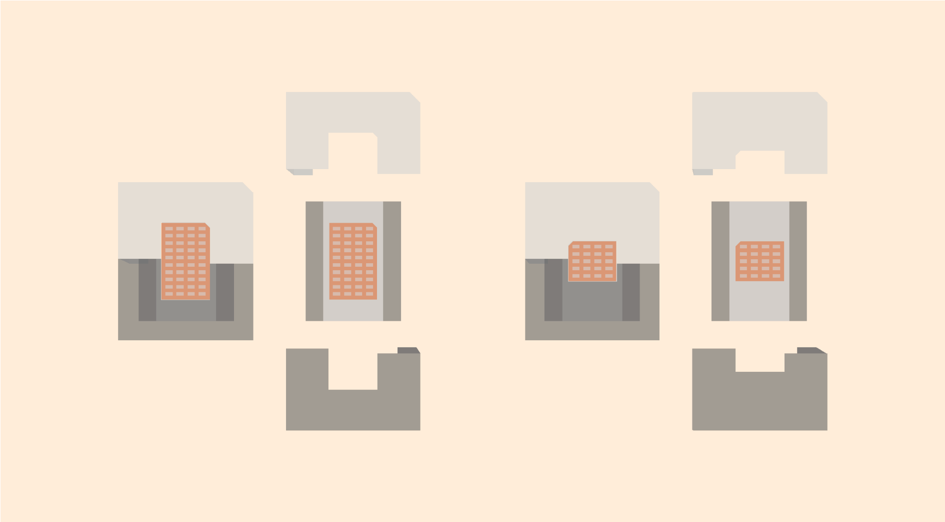

Top views of the plate adapters, open and locked, for both plate types: `plate_locker_top` *, the* `plate_holder` *with an assembled plate on top, and* `plate_locker_bottom` .

The plate holder is shared between all adapters, while lockers are specific for each plate type:

- `r5_c4_locker_top`
- `r5_c4_locker_bottom`
- `r10_c4_locker_top`
- `r10_c4_locker_bottom`

TRAY

The tray slides in and out of the `dark_box` ; thanks to the wall inclination, a rail below, and the top tab, no light will leak into the interior of the dark box.

Thanks to the dent on top, the full plate adapter once locked can only be put in the tray in the right orientation.

3.2.4 Parts List

Here number refers to how many copies of that part a single VPCIReader includes; it has 26 types of parts and 34 physical pieces.

Name	Component	Number	Color
box	Electronics Box	1	orange
box_top	Electronics Box	1	white
ventilation_grid	Electronics Box	1	orange
battery_right_tab	Electronics Box	1	white
battery_back_tab	Electronics Box	2	white
pi_board_separator_1	Electronics Box	1	white
pi_board_separator_2	Electronics Box	1	white
pi_board_holder	Electronics Box	1	white
fan_holder	Electronics Box	1	white
microUSB_holder	Electronics Box	1	white
power_button	Electronics Box	1	white
front_cover	Electronics Box	1	black
camera_box_bottom	Electronics Box	1	black
camera_box_top	Electronics Box	1	black
camera_inner_tube	Electronics Box	1	black
dark_box	Dark Box	1	black
dark_box_cover	Dark Box	1	white
bottom	Dark Box	1	orange
tray	Dark Box	1	black
plate_assembler_r5_c4	Plate Tools	2	black
plate_assembler_r10_c4	Plate Tools	2	black
plate_holder	Plate Tools	2	black
plate_locker_top_r5_c4	Plate Tools	2	white
plate_locker_bottom_r5_c4	Plate Tools	2	black
plate_locker_top_r10_c4	Plate Tools	2	white
plate_locker_bottom_r10_c4	Plate Tools	2	black

Appendix A

Electronics Setup

All the electronic components can and should be checked and configured *before* assembly. The different components can be set up in the order laid out here.

A.1 Battery

MATERIALS

- Voltaic V25 Battery
- microUSB to USB-C adapter
- USB-A to USB-C cable
- Raspberry Pi Power Source
- Raspberry Pi Board

STEPS

1. Plug the adapter into the USB-C charging port of the battery
2. Fully charge the battery using the power supply[1]
3. Plug the USB-A end of the cable into the battery, and the other USB-C end into the Raspberry Pi board. Check that the LEDs turn on.
4. Plug and unplug the power supply, turn the Raspberry Pi on and off with the battery button, etc.

[1]Batteries are normally shipped half charged; the battery will be fully charged once the 4 lights are continually on.

NOTES

- Fully charging an empty battery takes approx 1h.
- Do not plug a USB drive, insert an SD card, etc into the Raspberry Pi board; at this stage we just want to check that power works correctly.

A.2 Raspberry Pi

MATERIALS

- USB Drive
- Raspberry Pi Board
- power supply
- ethernet cable and wired network
- computer connected to the same network with propellor and secret keys

STEPS

1. Write the custom OS image to the USB drive
2. Plug the drive in one of the Pi USB3 ports[2]
3. Fix the heatsink to the microprocessor
4. Connect the Ethernet cable
5. Connect the Pi to the battery using the USB-A to USB-C cable, with the battery in turn connected to the power supply
6. Wait approx 5 minutes n order to give time for the first boot setup to finish, and reboot the Pi
7. Map DNS at the local computer (using for example `/etc/hosts`)
8. Spin the host using propellor from the local computer

NOTES

- Given the limited power of a Raspberry Pi, propellor bootstrapping and execution, including compiling the image analysis executables, will take around 5-6h. All the Pis part of a batch should then be set up in parallel.

[2]these are the ones with the blue plastic piece inside.

- The Pis should be in a cool room.

A.3 Camera

MATERIALS

- Fully set up Pi board
- Battery and power supply
- Camera
- Camera focus sample (PCB board, electronics with small text)
- Camera lens tool

STEPS

1. Connect the camera to the board, then turn it on.
2. Place the camera on a support with the focus sample at the correct distance.
3. Stream video from the Pi to an auxiliary device, and use the focus tool to slowly adjust the camera focus.
4. Once it looks good enough enough, start taking full resolution pictures and adjusting slowly between them.

NOTES

- Cameras are especially sensitive to static discharge; follow standard ESD precautions when performing the above.
- A single board can be used to configure a batch of cameras sequentially.

A.4 Fan

MATERIALS

- Fully set up Pi board
- Battery and power supply
- Case fan

STEPS

1. With the board off, plug the GPIO pins following the case fan documentation.
2. Turn the board on, and check that the fan starts and keeps going until you turn the board off.

NOTES

1. Check each fan with its corresponding board.

VPCIR Reader User Manual

Definitions

Things you use but you can not create or modify:

Assay Types:

The administrator should create the assay types. Here you can choose which assay Type to use when defining an experiment. You can list the Assay Types but can not change them or create new ones. Every experiment should be connected to an assay type which in addition to other information has the relevant information regarding the size of the plate in which the experiment will be performed. This is crucial for the analysis of the results and should be coherent with the real world plate you are using.

Sample Types:

The administrator should create the Sample Types. You can list and choose a sample type when creating a sample, but you can not change or create Sample Types

---

Things you can Create and Modify:

Samples:

Samples are used in the definition of experiments. You can create and modify samples which are connected to a Sample Type

Experiments:

You can create and modify new experiments. An Experiment depends on the Assay Type you choose when you create the experiment. The assay type will dictate the size of the plate. You should take care to be coherent with the real plate you are using. The size of the plate will give you N wells. You should define each well sample and the dilution of it. In addition you can change the default name and comments of each new experiment or change them later. When you create a new experiment it is created with default content (samples and dilution). If you are in a hurry you can read the experiment and change samples and dilutions later although this is not recommended. You can create an experiment as a “copy” of an old one. This could accelerate the definition of the experiment.

Reads:

A read is done over a previous experiment. When you decide to do a new read you should choose an experiment and then follow the process of reading in different screens: black picture, pictures of the plate, adjustment of the area to analyse and making the analysis. After all these steps you get the results of the reading corresponding to each picture of the ten pictures the reader takes.

Reports:

A Report is the results of one picture of a reading. When you are browsing the different pictures of a read you can always create a report corresponding to this specific frame

Listing and Searching

List is for obtaining ALL the items you can use. Search is to get the subset fulfilling the text criteria used. The search is done in name and comments fields. The result of a search is a list and behaves identical to a list, same icons and clicking possibilities. In general clicking the icon of an item in a list leads you to the edition of this item

New Items

As a general rule, when you created a new item this is created with default content and you can from this moment edit it

When you go to the URL: http://yourreader.local/ you see the login screen:

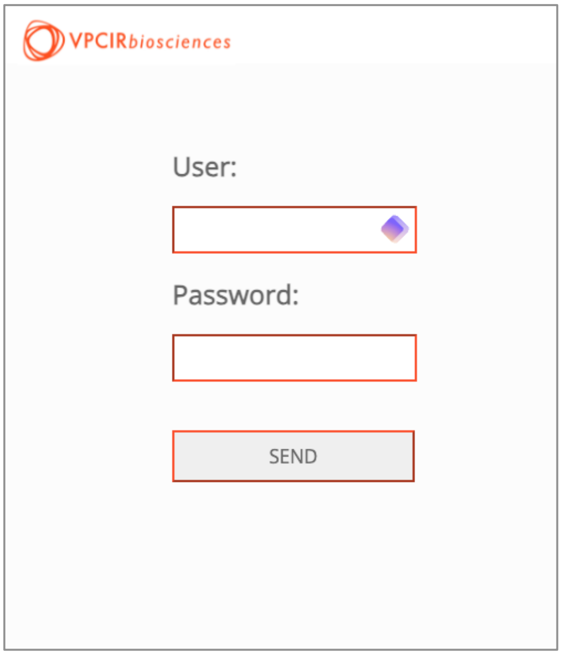

Once you introduce your username and password you go to the home screen:

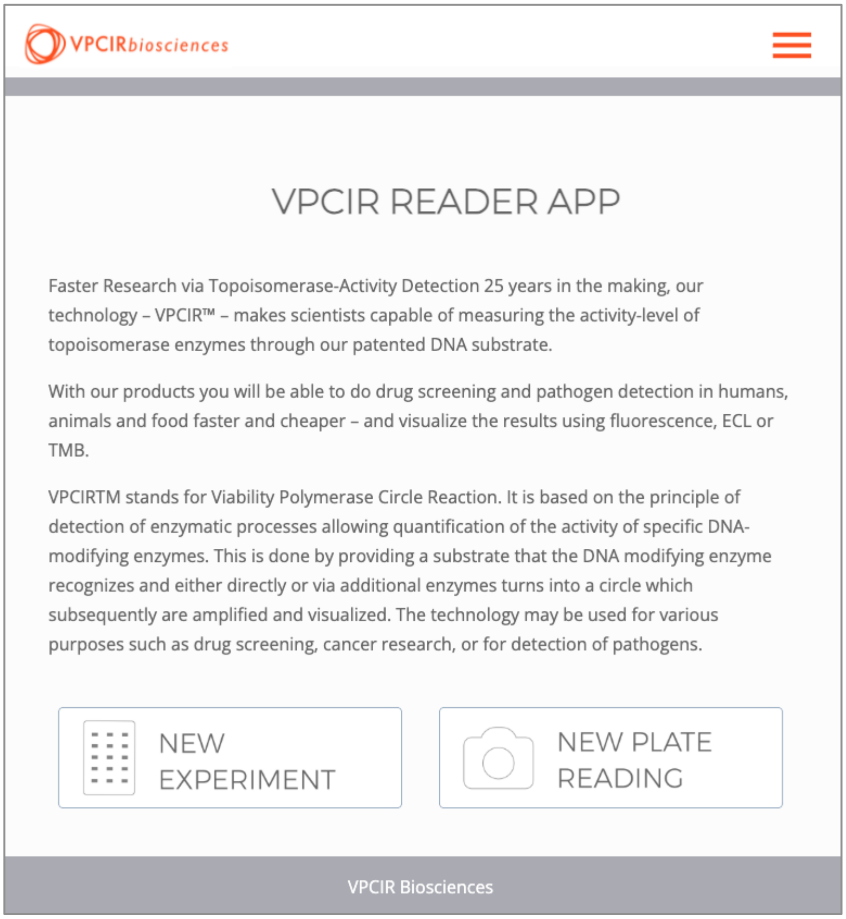

At the Home screen you can click the general menu icon (top right corner) or use two icons intended to be shortcuts for "NEW EXPERIMENT" and "NEW PLATE READING"
You can do that also from the Experiments and Plate Reads submenus.

Menu Options

If you click the general menu icon you get the following options:

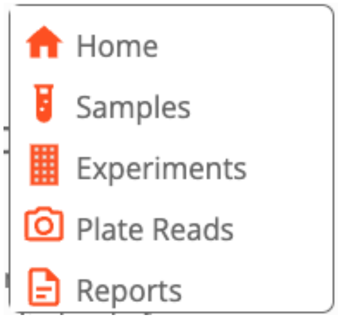

- Back to the Menu screen
- Samples
- Experiments
- Plate Reads
- Reports

Samples

When you click the Samples Option in the general Menu you are directed to the samples submenu:

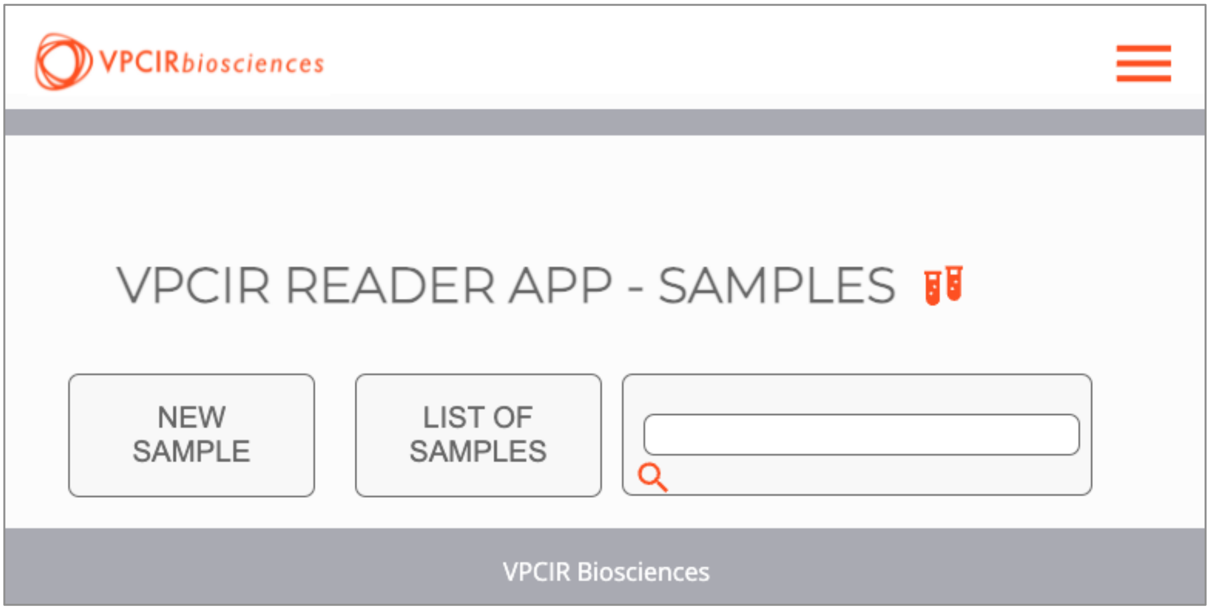

Listing Samples

You can click on “LIST OF SAMPLES” and get all the samples you can use:

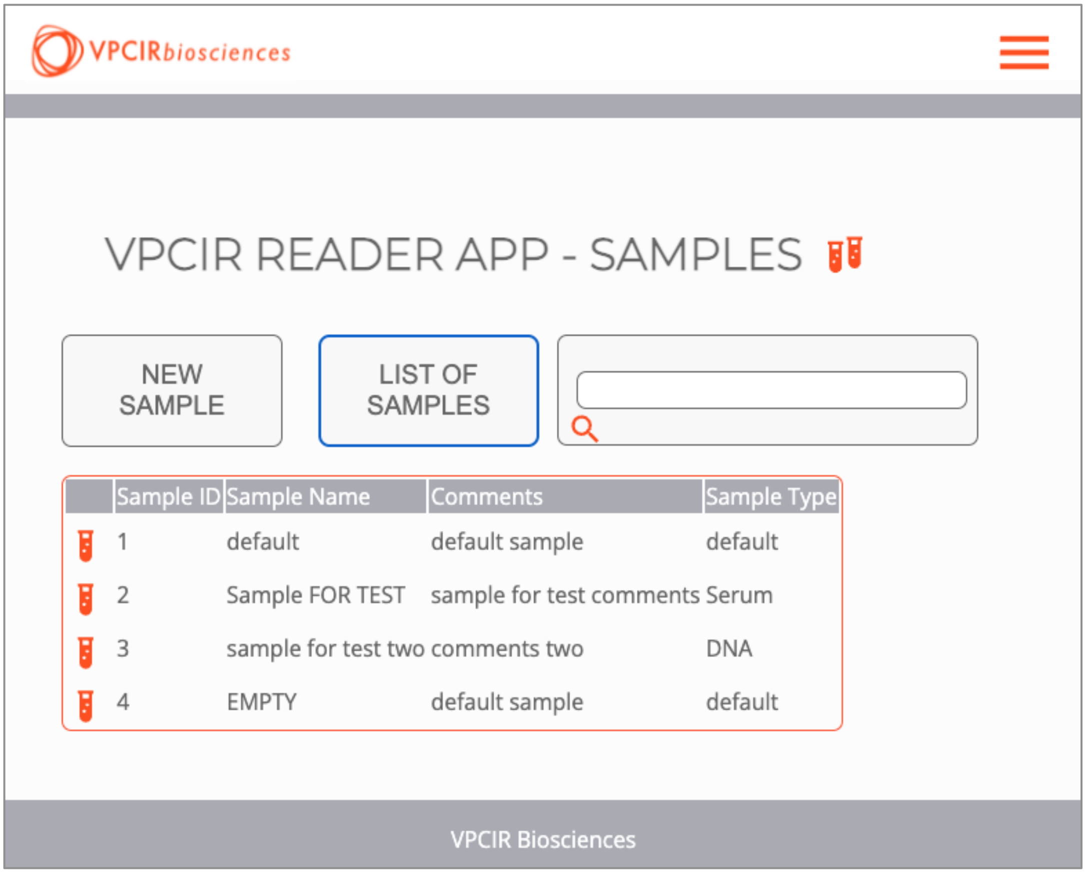

When you search you obtain a list of samples. In any case you can get more info of a sample and edit it clicking the tube icon:

View / Editing a Sample:

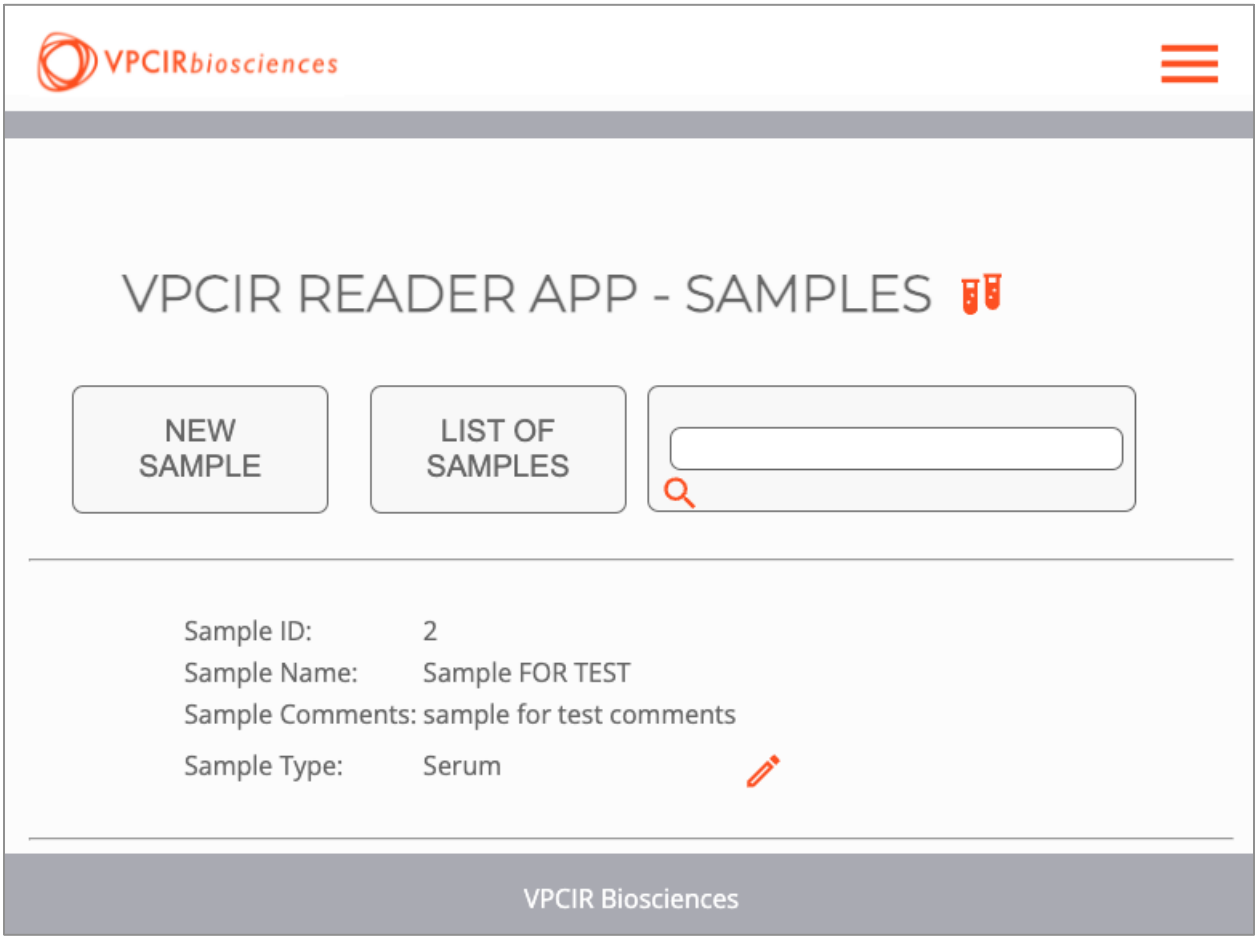

Clicking the pen icon you can edit the info of the sample. Notice that , in addition that the name and comments field become editable, you also have a list of sample types to change if you want the sample type of this sample:

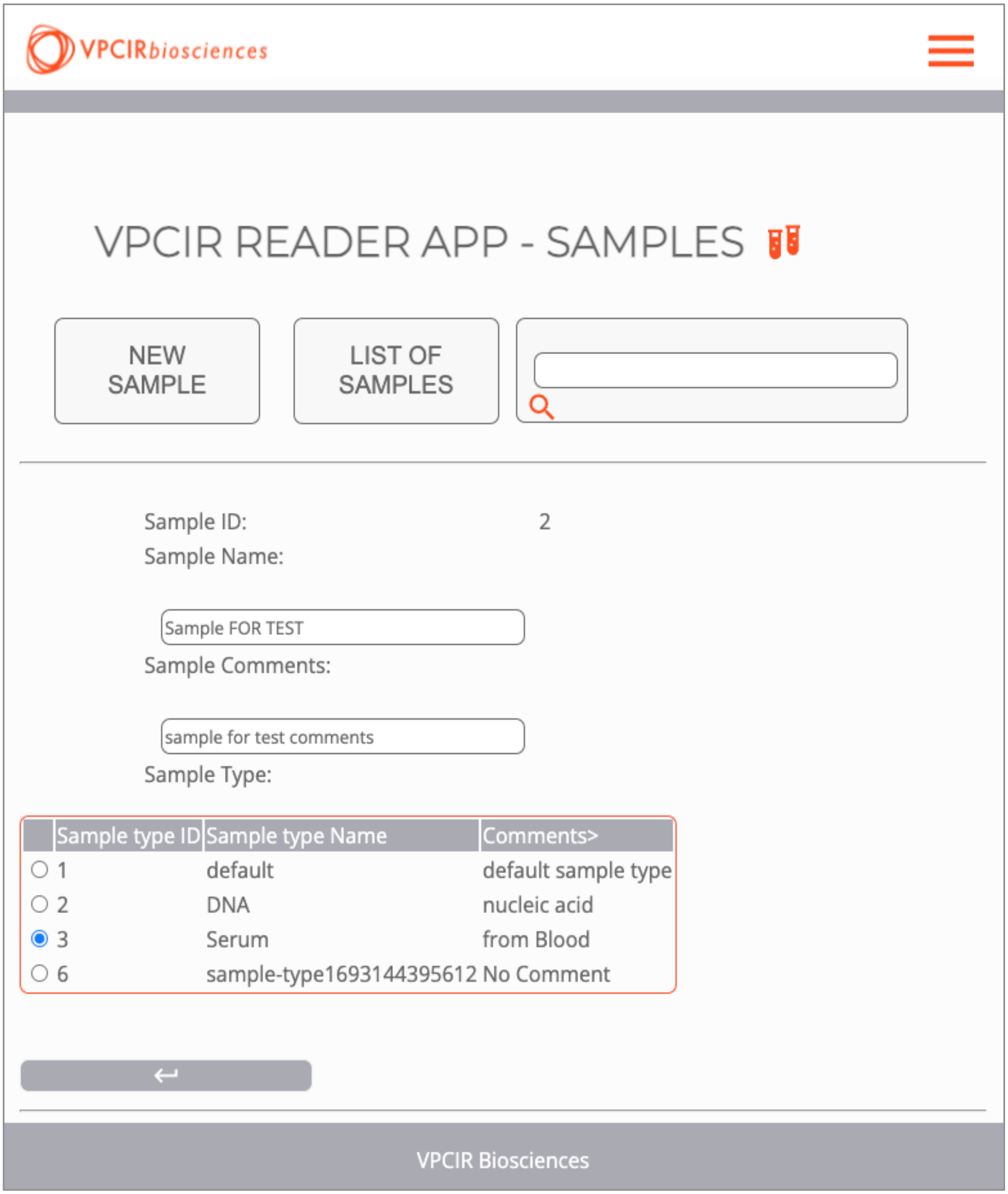

Once you change the things you want to change you should press the “enter” button to get the changes done

New Sample

If you click in the “NEW SAMPLE” option of the samples submenu you get this screen:

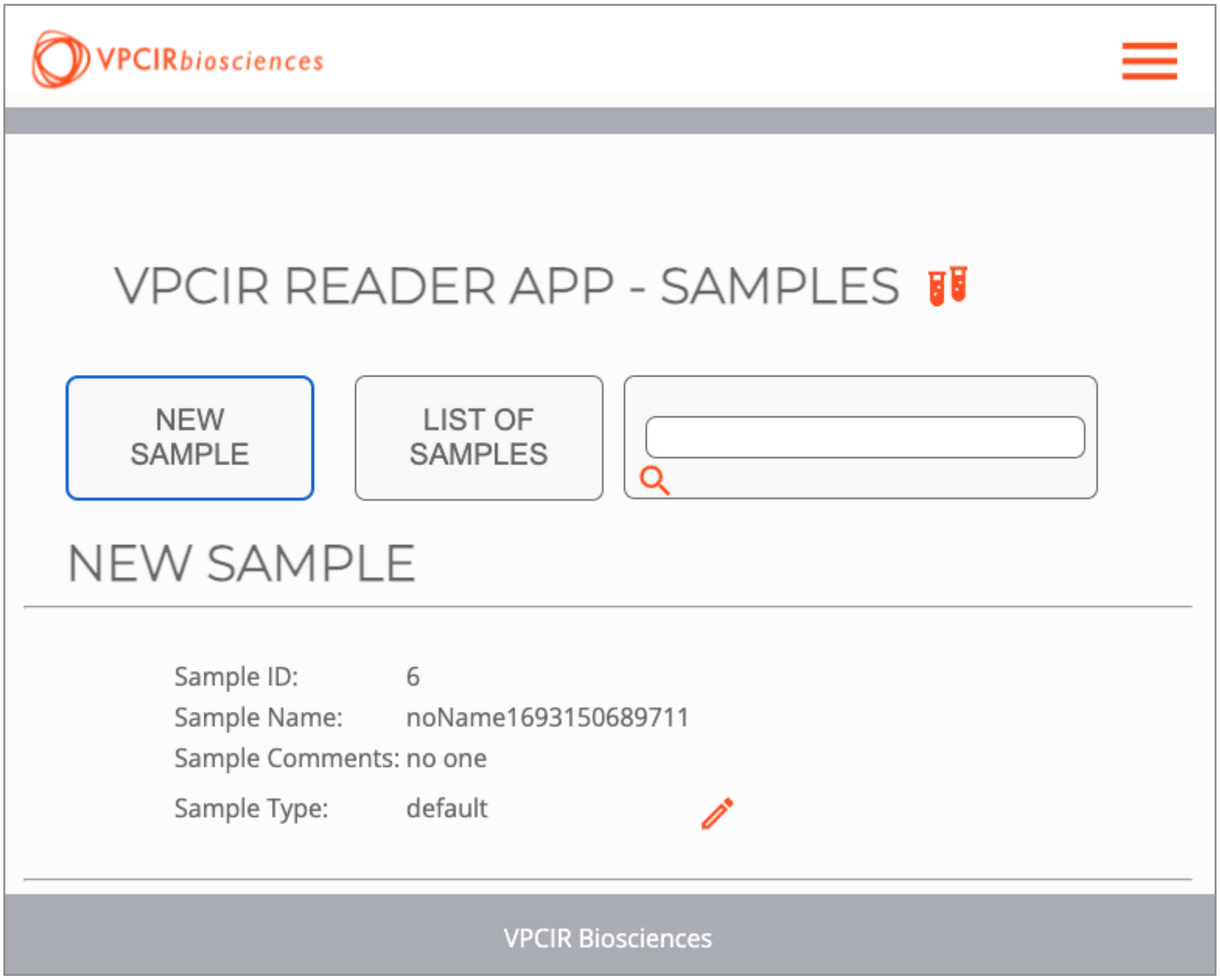

This means that the new sample has been created with default content. Now you can press the pen icon to go to the edit screen and change all the things you would want to change.

Experiments

When you click Experiments Option in the general menu you get the Experiments submenu:

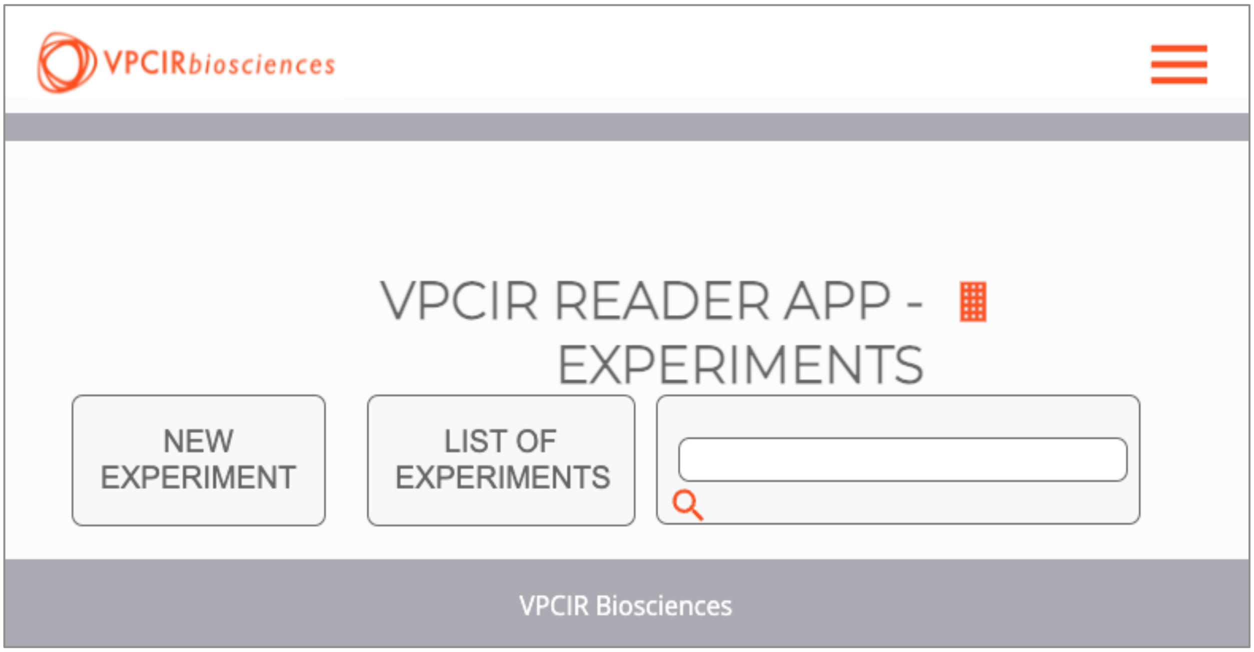

Listing Experiments

When you click the “LIST OF EXPERIMENTS” option you get the list of ALL the experiments you can use / edit . If you search experiments you will get a subset with the experiments having the text used to search in its name or comments

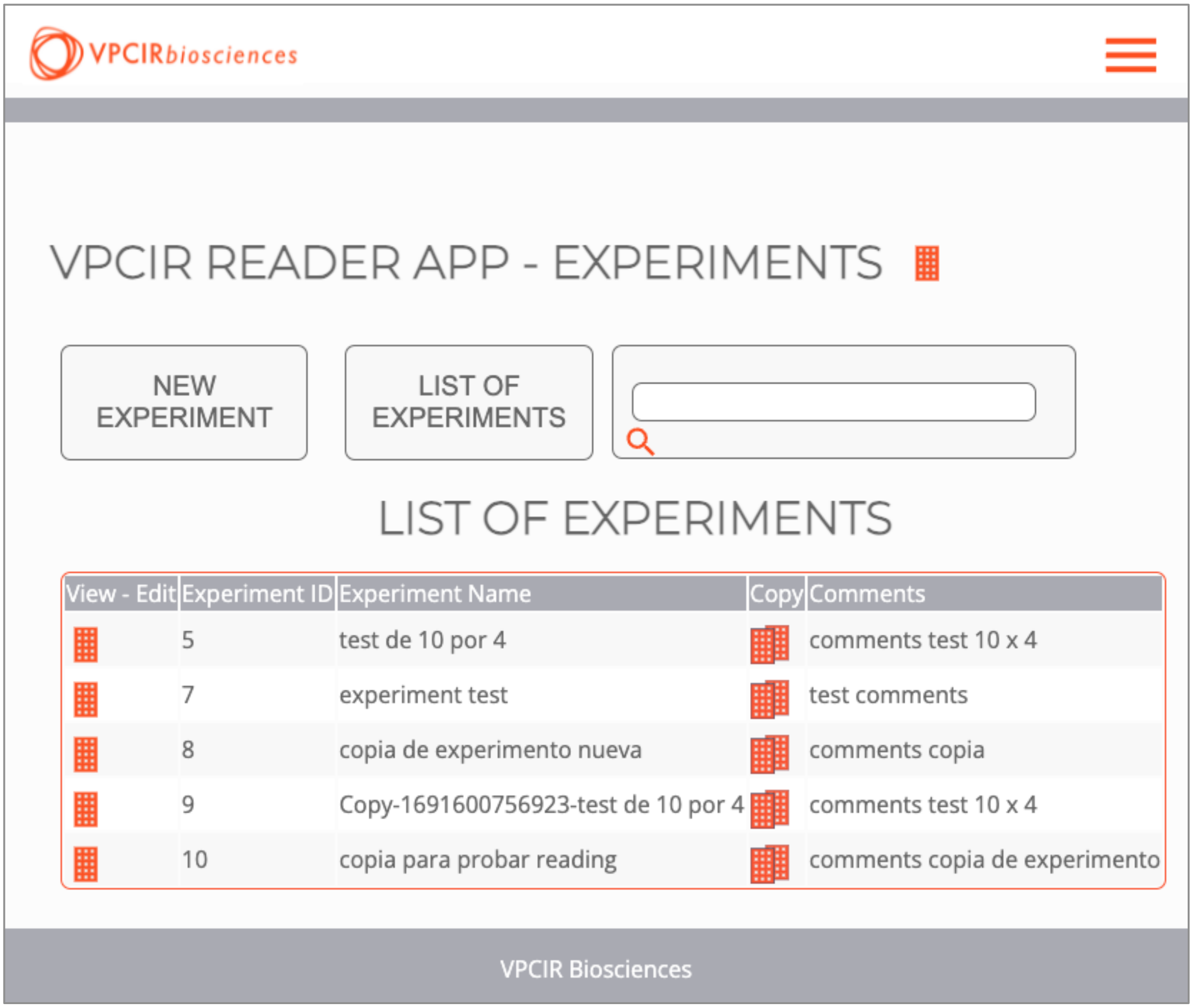

If you click the single plate icon you will go to the view / edit experiment screen.

If you press the two plates icon you will get A NEW EXPERIMENT with the same data as the existing experiment but with the term “copy” added to its name.

This is a convenient way to facilitate the definition of an experiment

View / Edit Experiment:

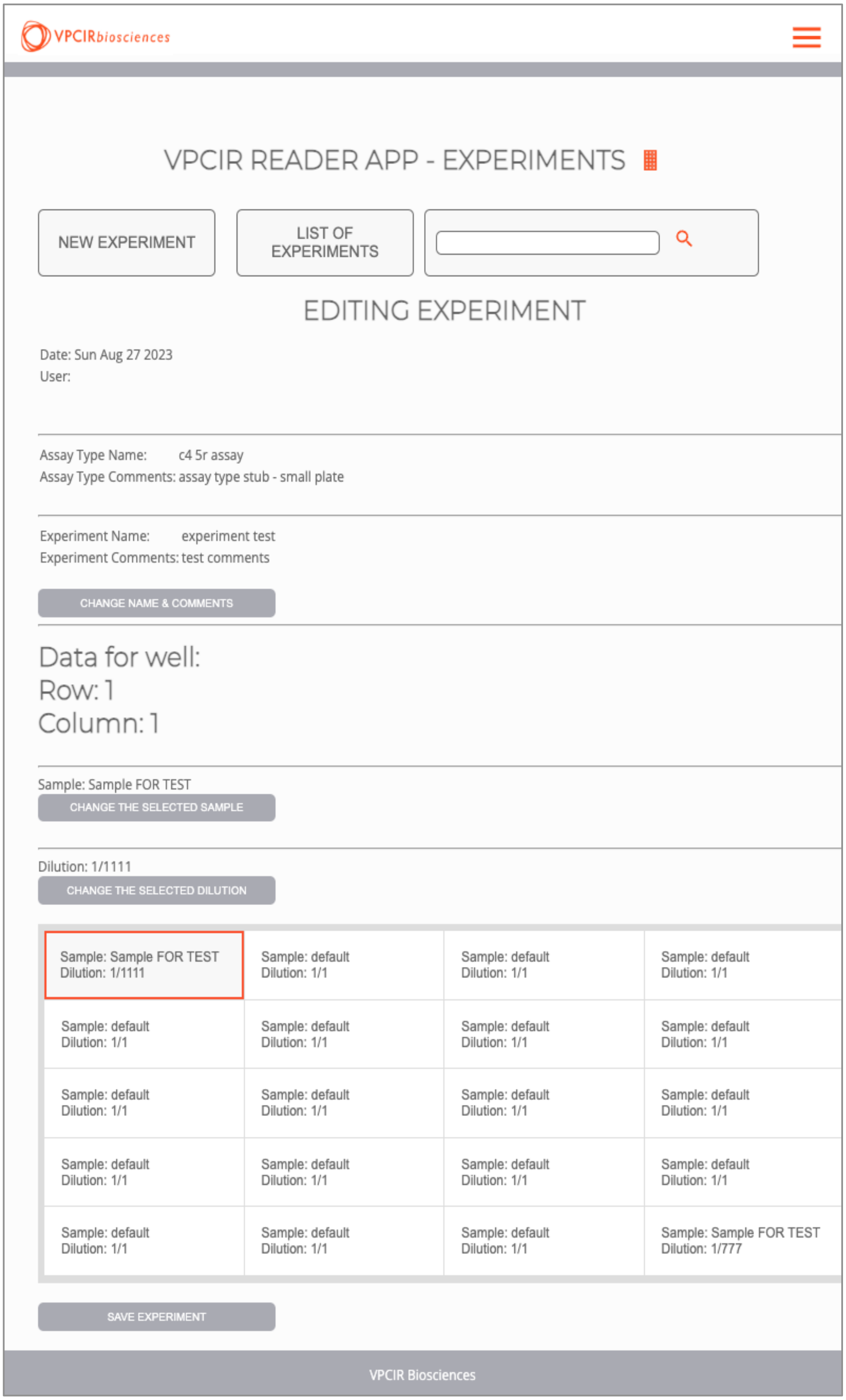

When you click the plate icon in an experiment in a list of experiments you get the edit screen shown above.

- First data that you can not change as Assay Type (you can create a new one with a different assay type
- Then the name and comments of the experiment that you can change. For that you should press the change name and comment button and these fields become editable:

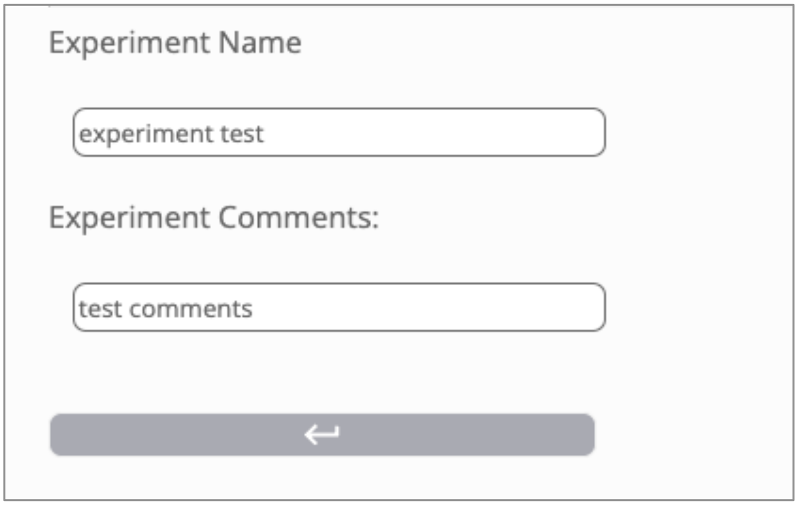

- Then you have the data for the selected well. By default the row 1 column 1 well. You can press another well in the grid below to edit its content. Once selected the well you want to change, press "CHANGE THE SELECTED SAMPLE" to change the sample for this well and this will become editable.

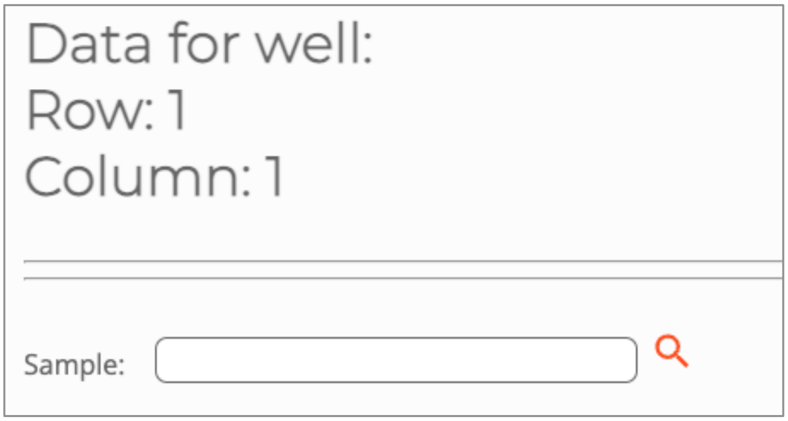

- A search field will appear and you can enter some text or leave the field empty and press the magnifier . In this case you will obtain all the available samples:

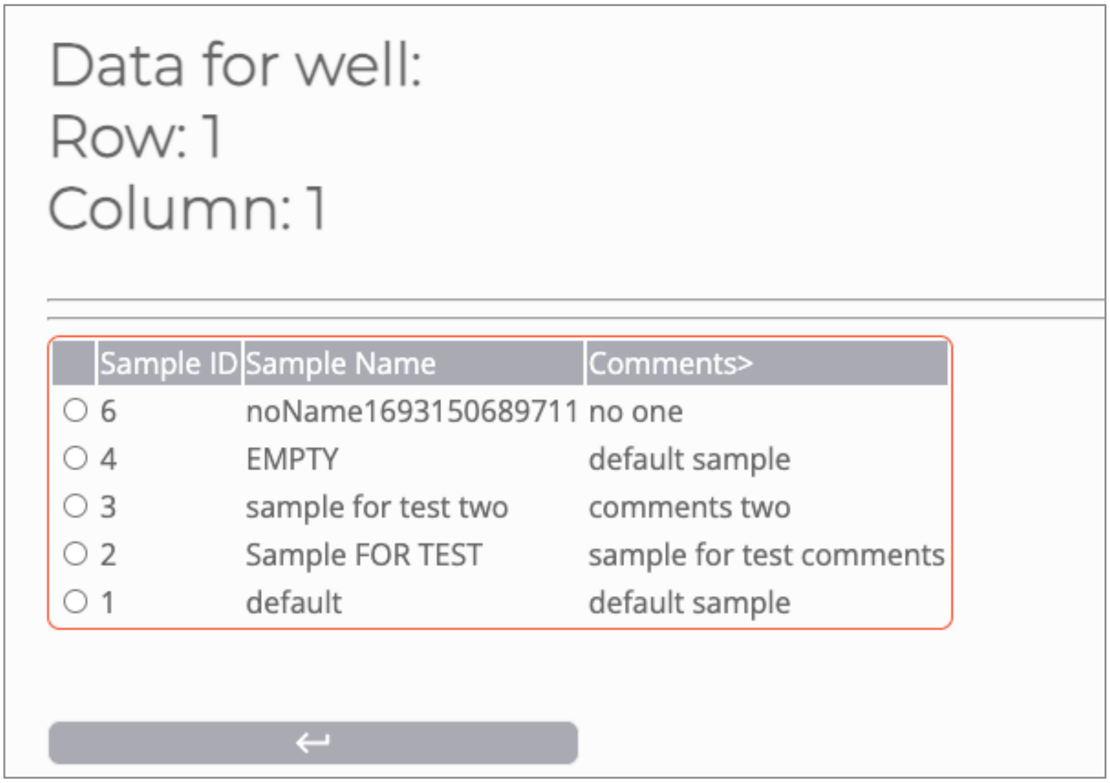

After choosing a sample for this well you should press the “enter” button

- Then you have the dilution for this well and sample. You can change it by pressing the CHANGE THE SELECTED DILUTION button. Once pressed you get the dilution editable:

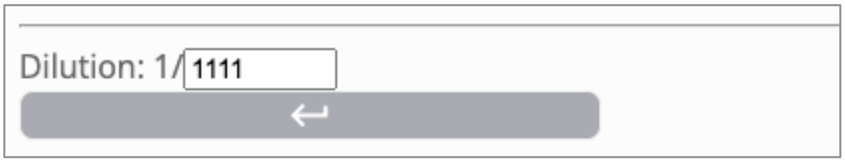

Again you should press “enter” button to change the dilution with the desired value

- Then you have the grid with all the wells. Clicking a well will select this well and a red border will indicate that it is the selected one:

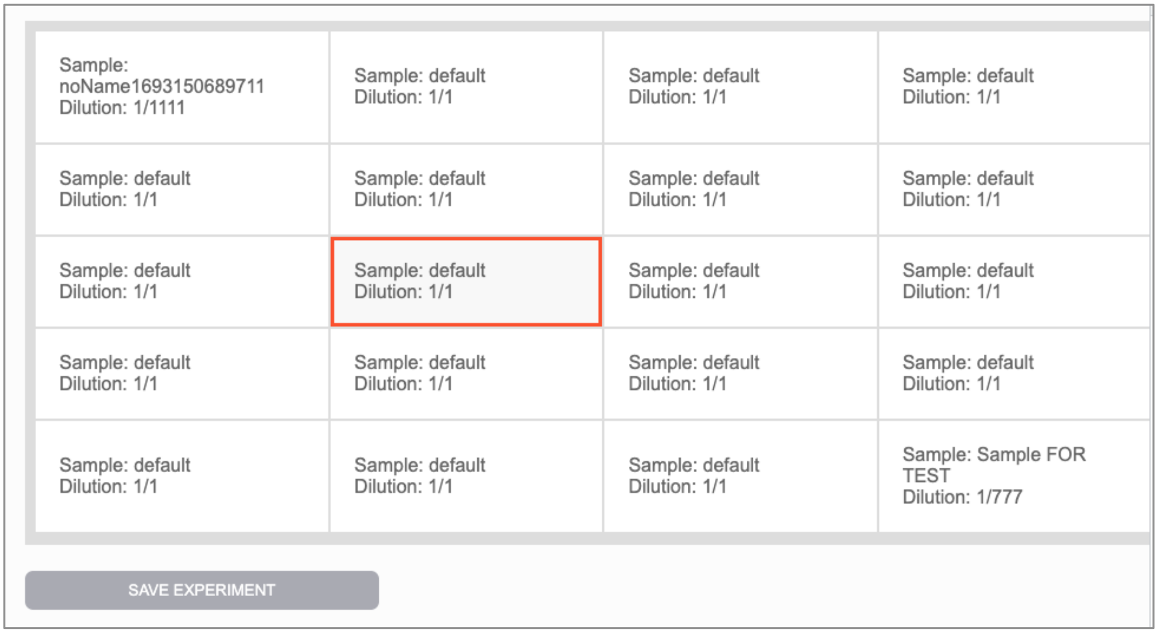

After completing all changes do not forget to click “SAVE EXPERIMENT” button at the bottom of the page.

If all is OK you will get an alert communicating that the experiment has been saved:

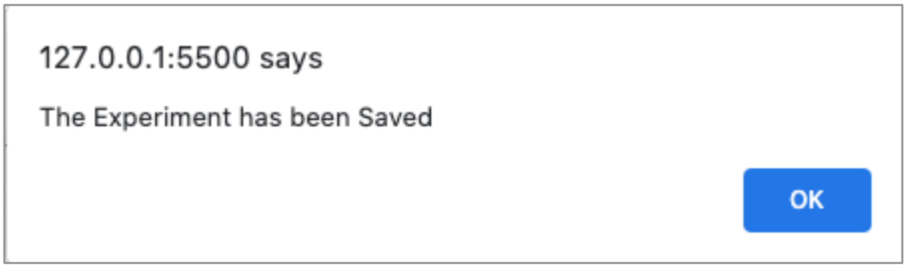

Note that URL before “says” will change depending on the reader.

New Experiment

Clicking the “NEW EXPERIMENT” option in the experiment submenu or using the shortcut button of the home screen, firstable you will be asked to choose the assay type for this new experiment. Remember that this is important as this option will determine the number of rows and columns (the type of plate) and this will not be possible to change:

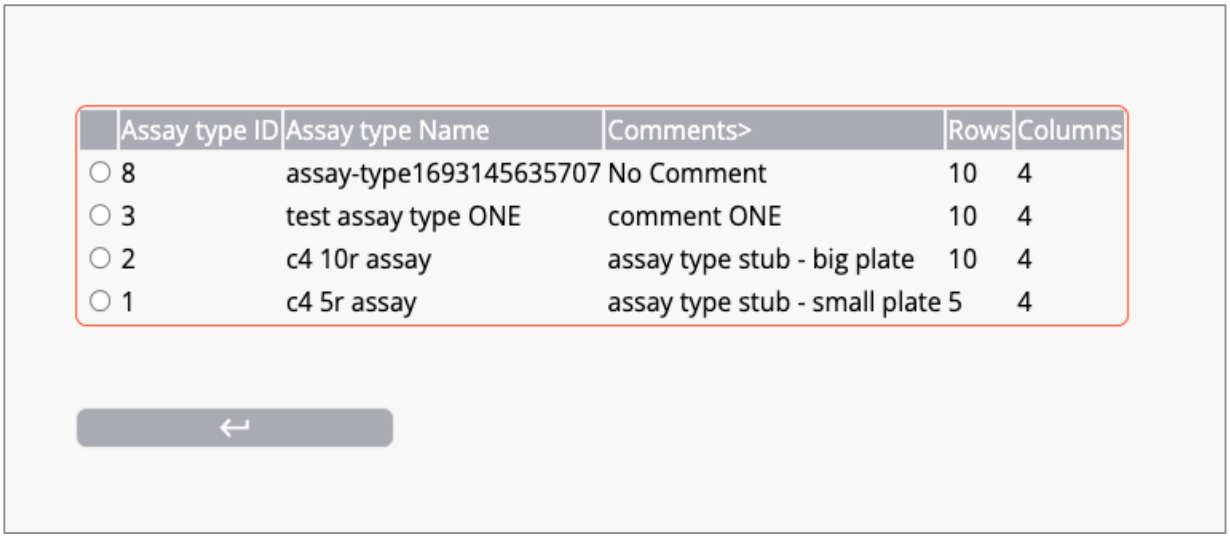

	Assay type ID	Assay type Name	Comments>	Rows	Columns
○	8	assay-type1693145635707	No Comment	10	4
○	3	test assay type ONE	comment ONE	10	4
○	2	c4 10r assay	assay type stub - big plate	10	4
○	1	c4 5r assay	assay type stub - small plate	5	4

Remember to press the “enter” button once you select the assay type. Once pressed you will get the edit experiment screen with all the default values and now you have the possibility of editing it as we have seen above:

VPCIRbiosciences

VPCIR READER APP - EXPERIMENTS

NEW EXPERIMENT | LIST OF EXPERIMENTS

EDITING EXPERIMENT

Date: Sun Aug 27 2023
User:

Assay Type Name: c4 5r assay
Assay Type Comments: assay type stub - small plate

Experiment Name: Untitled-1693155226761
Experiment Comments: Still no comment

CHANGE NAME & COMMENTS

Data for well:
Row: 1
Column: 1

Sample: EMPTY
CHANGE THE SELECTED SAMPLE

Dilution: 1/1
CHANGE THE SELECTED DILUTION

Sample: EMPTY Dilution: 1/1	Sample: EMPTY Dilution: 1/1	Sample: EMPTY Dilution: 1/1	Sample: EMPTY Dilution: 1/1
Sample: EMPTY Dilution: 1/1	Sample: EMPTY Dilution: 1/1	Sample: EMPTY Dilution: 1/1	Sample: EMPTY Dilution: 1/1
Sample: EMPTY Dilution: 1/1	Sample: EMPTY Dilution: 1/1	Sample: EMPTY Dilution: 1/1	Sample: EMPTY Dilution: 1/1
Sample: EMPTY Dilution: 1/1	Sample: EMPTY Dilution: 1/1	Sample: EMPTY Dilution: 1/1	Sample: EMPTY Dilution: 1/1
Sample: EMPTY Dilution: 1/1	Sample: EMPTY Dilution: 1/1	Sample: EMPTY Dilution: 1/1	Sample: EMPTY Dilution: 1/1

SAVE EXPERIMENT

VPCIR Biosciences

Plate Reads

If you click “Plate Reads” in the general menu you go to the Reads submenu:

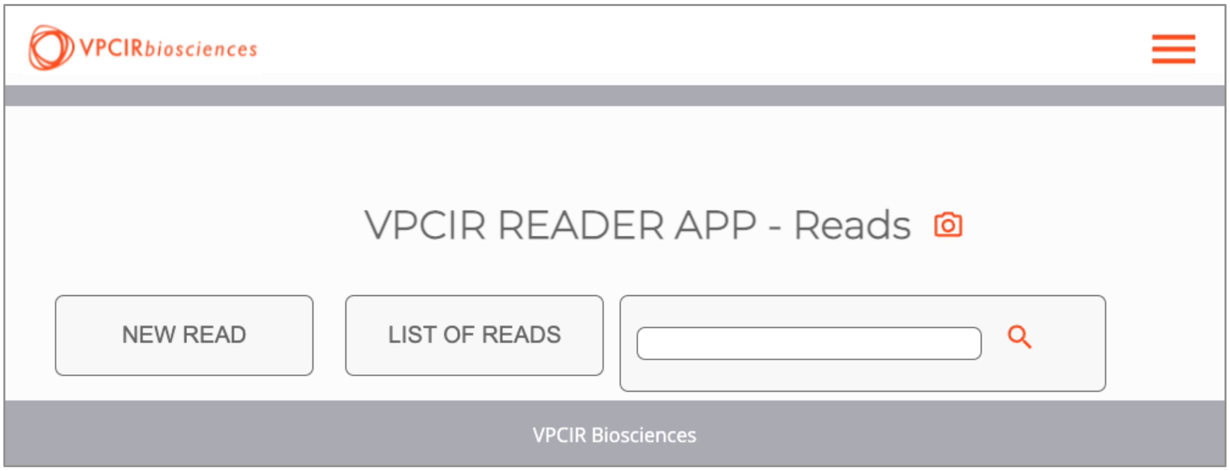

This submenu, as usual in this application, has three options: search , lint and new Read. As explained before, the search option produces a very similar output than the list option. The difference is that you get the list of the subset having in the name or the comments fields the text you want to search. In fact, a searching action without any text input lists ALL the reads available.

Listing Reads

If you click the List option you get a List of ALL the reads available. Once we have a list, either from search or from list option, the behaviour is identical: you can view / edit a read by clicking the camera icon of this row.

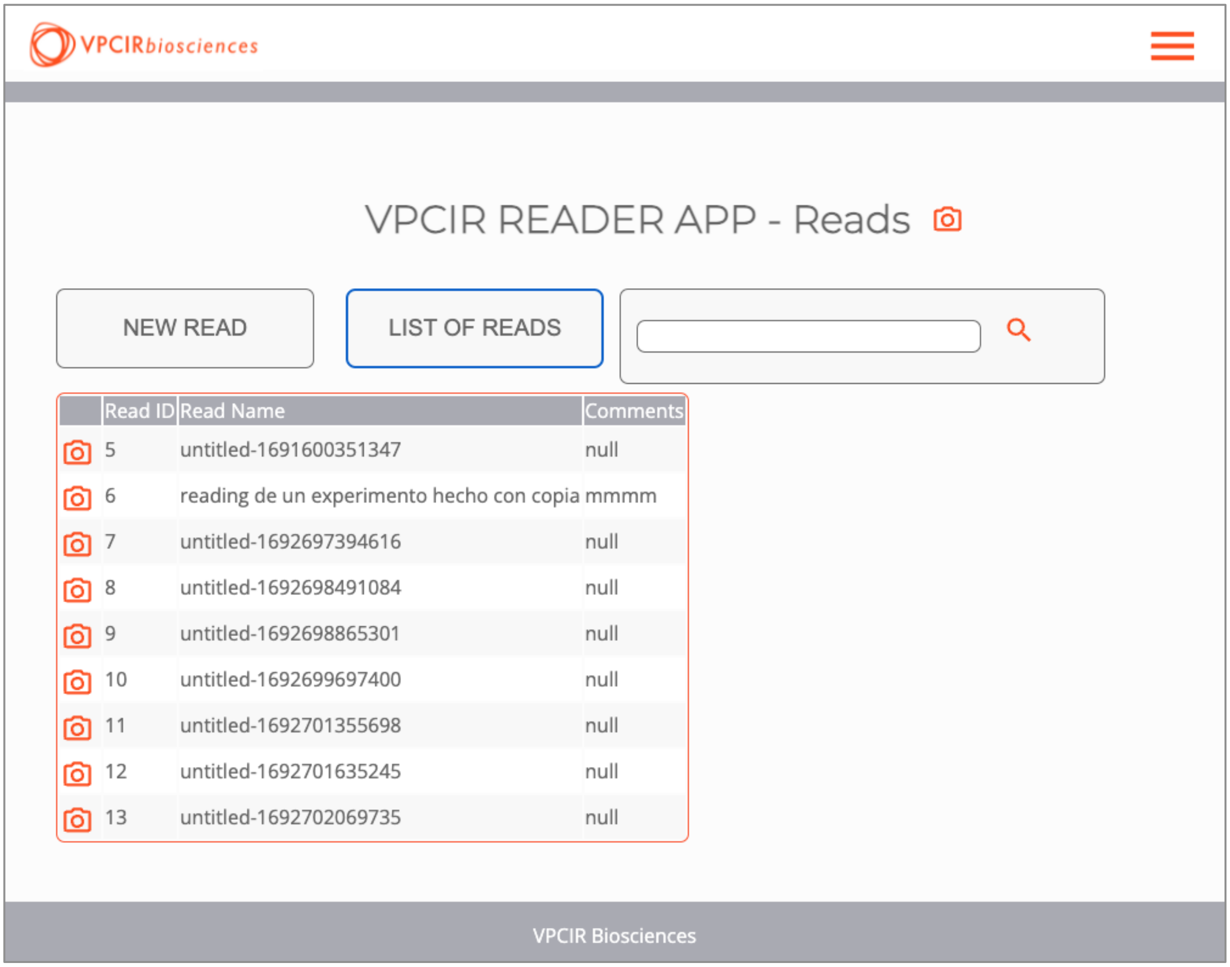
VPCIRbiosciences
VPCIR READER APP - Reads
NEW READ
LIST OF READS
Read ID
Read Name
Comments
5 untitled-1691600351347 null
6 reading de un experimento hecho con copia mmmm
7 untitled-1692697394616 null
8 untitled-1692698491084 null
9 untitled-1692698865301 null
10 untitled-1692699697400 null
11 untitled-1692701355698 null
12 untitled-1692701635245 null
13 untitled-1692702069735 null
VPCIR Biosciences

View / Editing A Read

When you click the camera icon in a list of reads you go to the view / editing read screen:
This is the upper part of the screen:

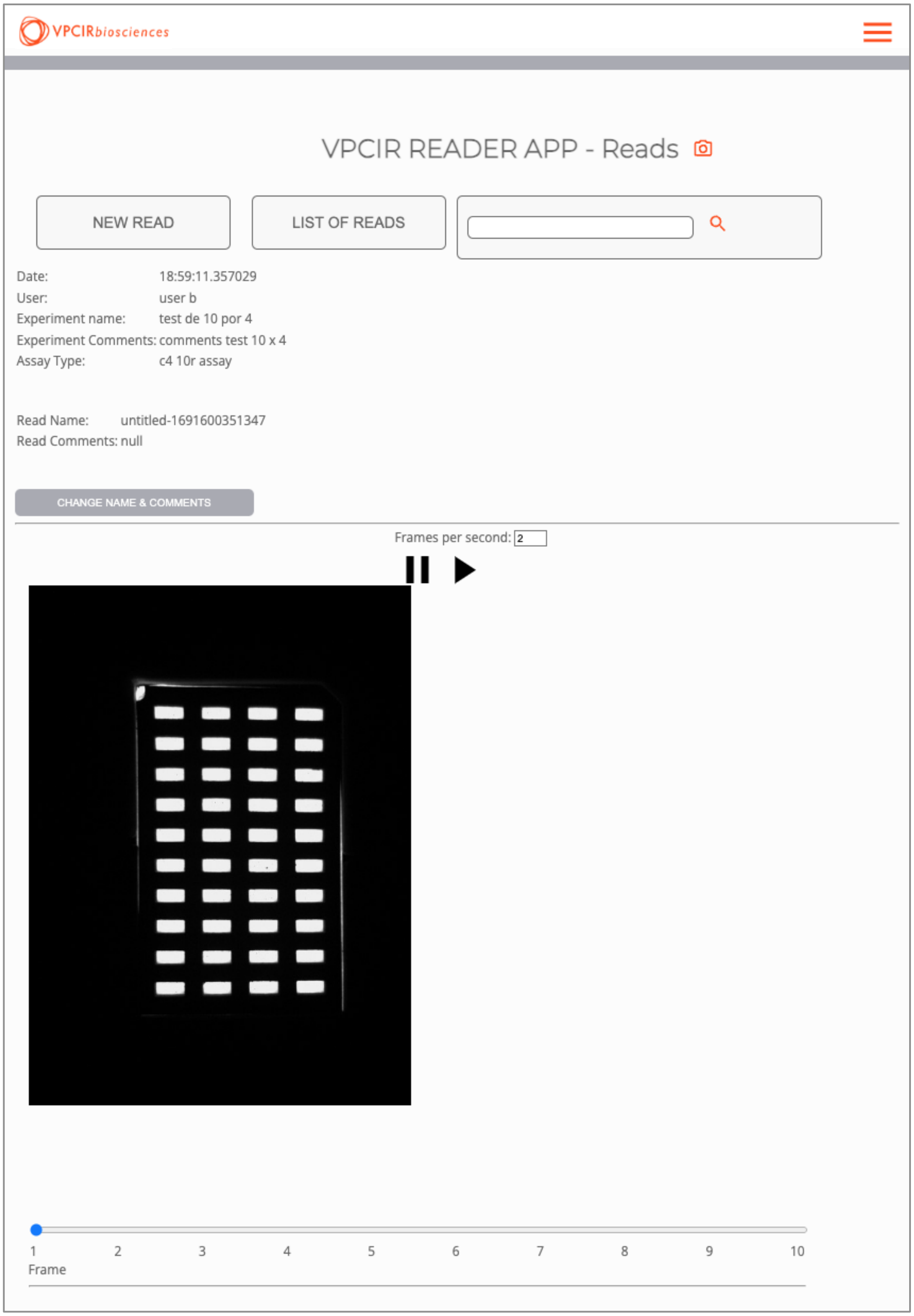

First you have The general data of the read and name and comments.
Here, you can edit the name and comments clicking the “CHANGE NAME AND COMMENTS” button

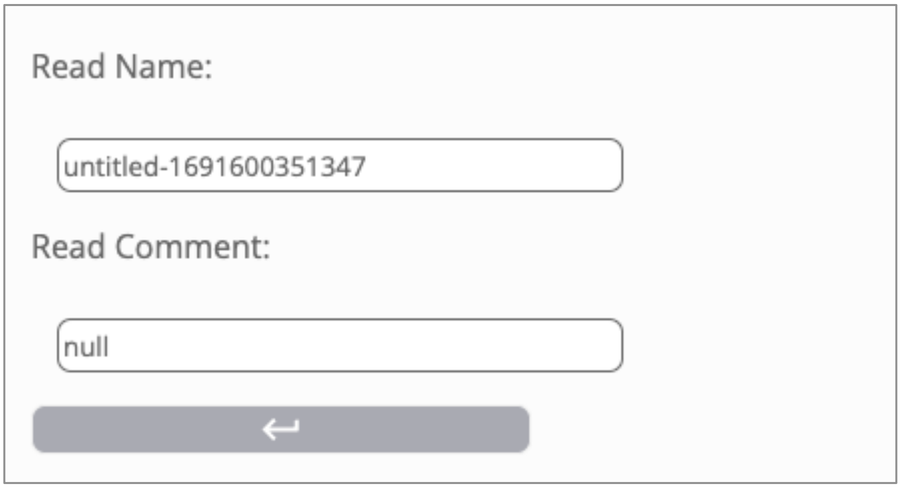

Once you have edited the name and comments press the “enter” button

A read is a series of ten pictures (frames) and their corresponding read values for each well of the plate.

In this screen you can navigate the ten frames in two modes:

- As a video clicking play button (you can use the pause button to stop at any moment). You can set the number of frames per second. By default it is set to two
- With a slider. In this case you navigate to the frame where you release the slider button.

In any case, navigating the frames you can see the corresponding picture and bellow a grid with the result for each well of the plate:

Frame

Sample: default<br>Dilution: 1/1<br>Signal: 7310	Sample: default<br>Dilution: 1/1<br>Signal: 6627	Sample: default<br>Dilution: 1/1<br>Signal: 5992	Sample: default<br>Dilution: 1/1<br>Signal: 5995
Sample: default<br>Dilution: 1/1<br>Signal: 6669	Sample: default<br>Dilution: 1/1<br>Signal: 6765	Sample: default<br>Dilution: 1/1<br>Signal: 6374	Sample: default<br>Dilution: 1/1<br>Signal: 6084
Sample: default<br>Dilution: 1/1<br>Signal: 6931	Sample: default<br>Dilution: 1/1<br>Signal: 6757	Sample: default<br>Dilution: 1/1<br>Signal: 6654	Sample: default<br>Dilution: 1/1<br>Signal: 6325
Sample: default<br>Dilution: 1/1<br>Signal: 6753	Sample: default<br>Dilution: 1/1<br>Signal: 6620	Sample: default<br>Dilution: 1/1<br>Signal: 6492	Sample: default<br>Dilution: 1/1<br>Signal: 6528
Sample: default<br>Dilution: 1/1<br>Signal: 6183	Sample: default<br>Dilution: 1/1<br>Signal: 6323	Sample: default<br>Dilution: 1/1<br>Signal: 6070	Sample: default<br>Dilution: 1/1<br>Signal: 5811
Sample: default<br>Dilution: 1/1<br>Signal: 5357	Sample: default<br>Dilution: 1/1<br>Signal: 5519	Sample: default<br>Dilution: 1/1<br>Signal: 5657	Sample: default<br>Dilution: 1/1<br>Signal: 5465
Sample: default<br>Dilution: 1/1<br>Signal: 4894	Sample: default<br>Dilution: 1/1<br>Signal: 5110	Sample: default<br>Dilution: 1/1<br>Signal: 5388	Sample: default<br>Dilution: 1/1<br>Signal: 5195
Sample: default<br>Dilution: 1/1<br>Signal: 5649	Sample: default<br>Dilution: 1/1<br>Signal: 5172	Sample: default<br>Dilution: 1/1<br>Signal: 4768	Sample: default<br>Dilution: 1/1<br>Signal: 5176
Sample: default<br>Dilution: 1/1<br>Signal: 5331	Sample: default<br>Dilution: 1/1<br>Signal: 5148	Sample: default<br>Dilution: 1/1<br>Signal: 5530	Sample: default<br>Dilution: 1/1<br>Signal: 5197
Sample: default<br>Dilution: 1/1<br>Signal: 5481	Sample: default<br>Dilution: 1/1<br>Signal: 5278	Sample: default<br>Dilution: 1/1<br>Signal: 5668	Sample: default<br>Dilution: 1/1<br>Signal: 5180

New Report from this Frame

At the bottom of the page there is an icon to create a report for the current frame.
Clicking this icon you create a report
An alert will inform you that the new report has been saved:

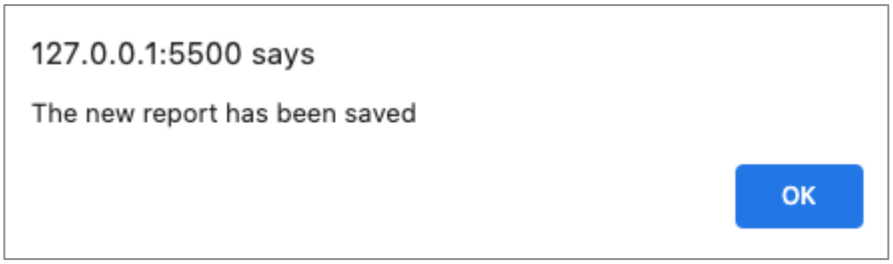

New Read

To get a new read you can press the “NEW READ” button of the read submenu or you can use the shortcut button for new read in the home page.

Once you click either button you are asked to search for experiments. Each read is made over an experiment. You can make several reads from the same experiment and each one will be a different read although linked to the same experiment

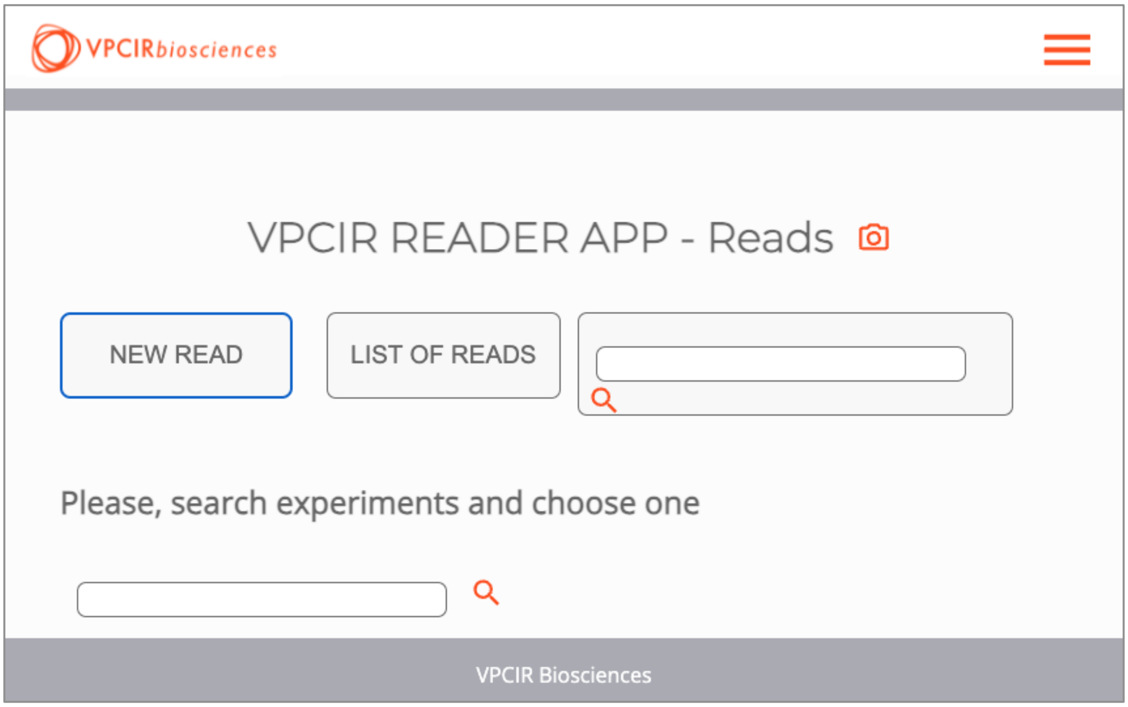

Once you enter a text and click the magnifier or simply click the magnifier without any text to search, you get a list of experiments where you should select one:

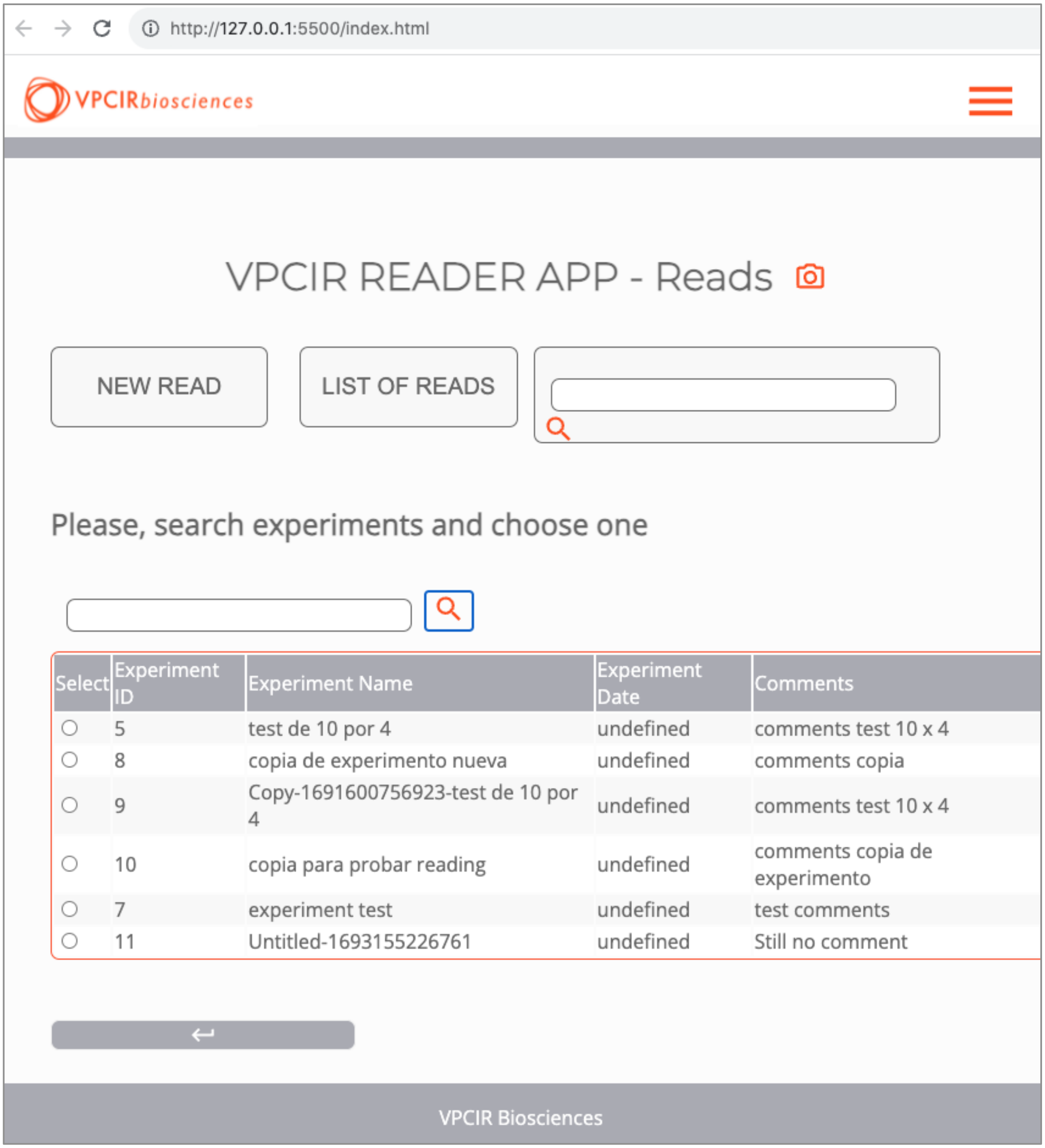

As always you should press the “enter” button once an experiment has been selected.

Once you select an experiment, the applications ask you to do a “black” picture: a picture without any plate inside the reader. This is the screen asking you to do that:

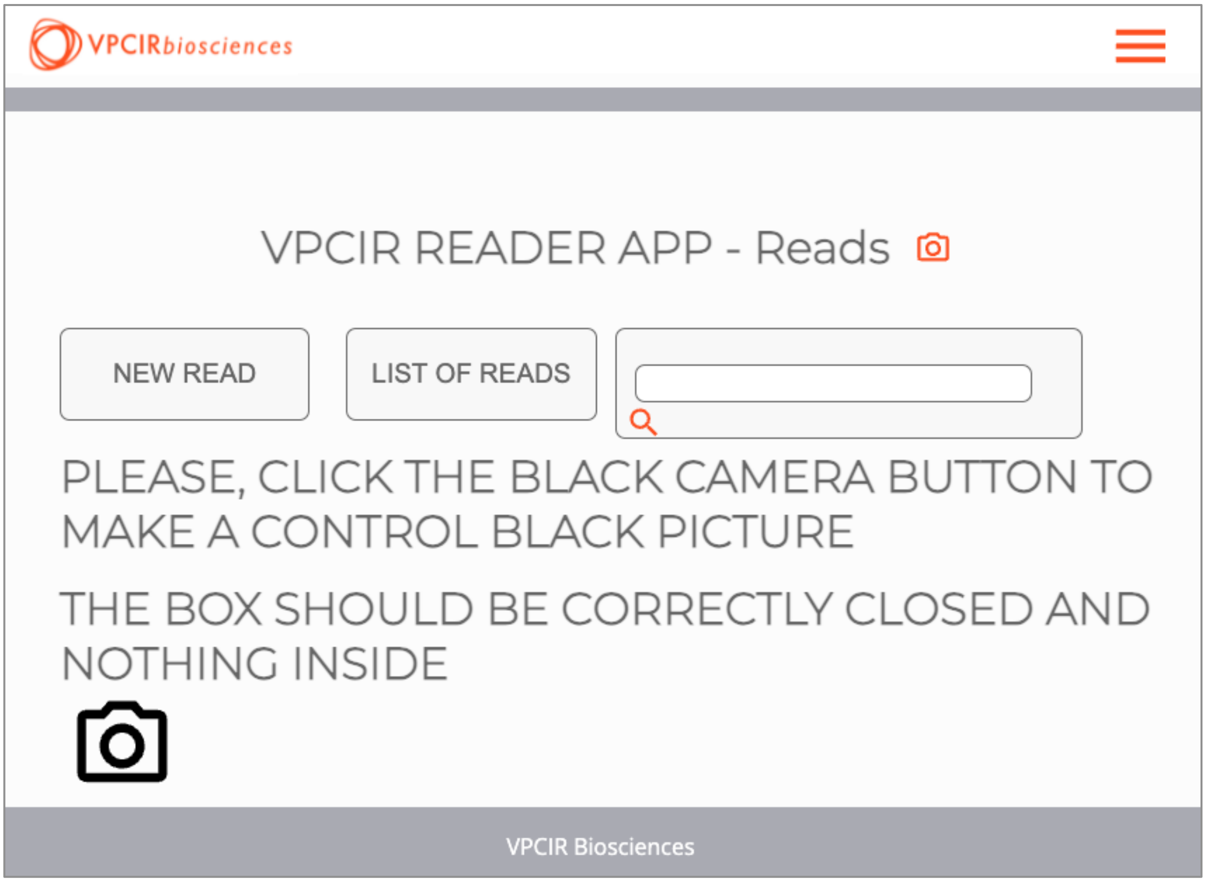

Once you are sure that no plate is inside the reader and that the tray is closed you should click the black camera icon. Once clicked, you get a wait screen:

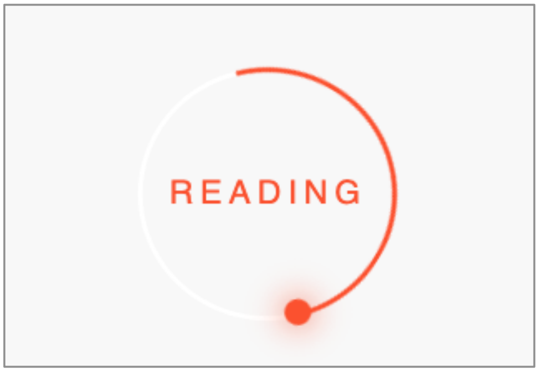

After a few seconds , the time to take a black picture, you are asked to put your plate inside the reader and close the tray:

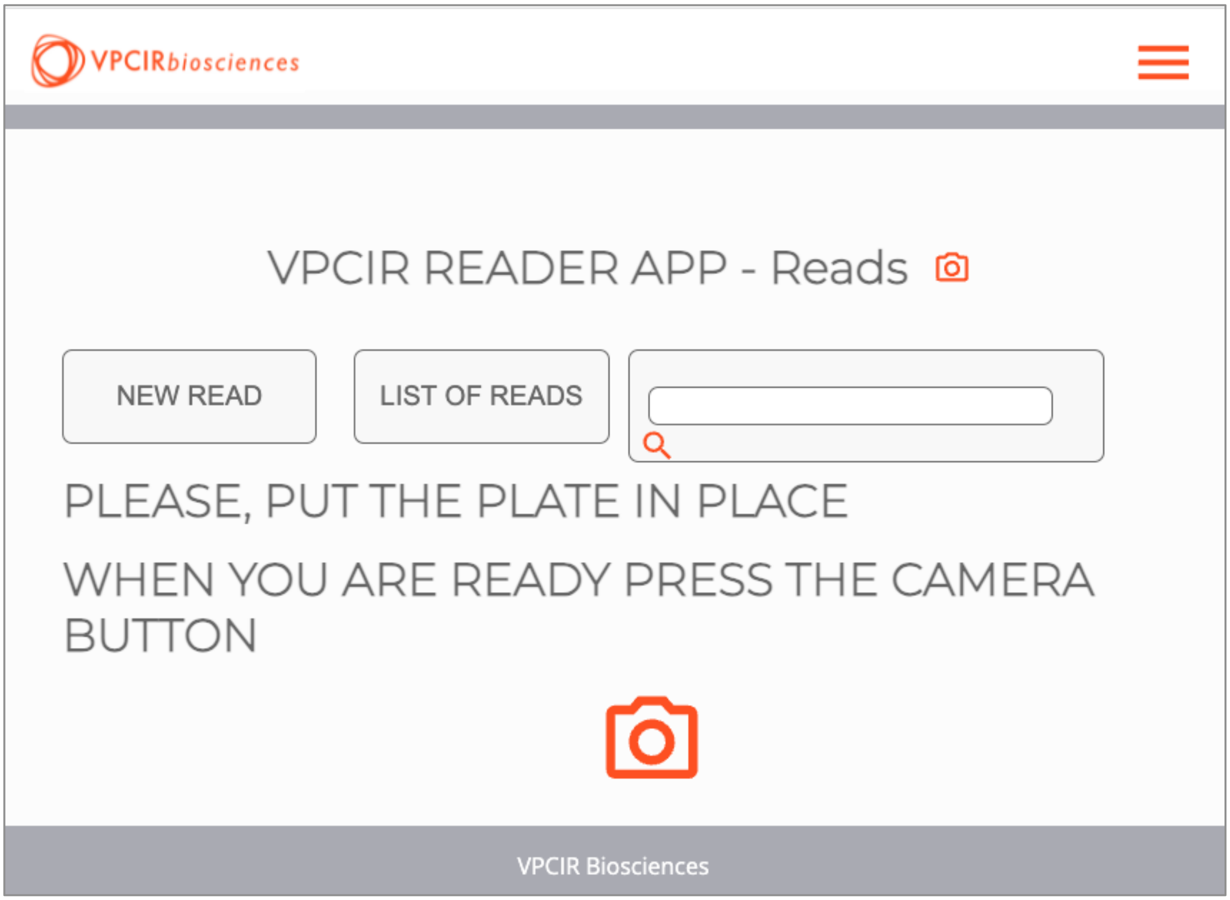

When you click the orange camera button you get the “READING” message again. This time it will be longer as the reader will take ten pictures of 10 seconds each.

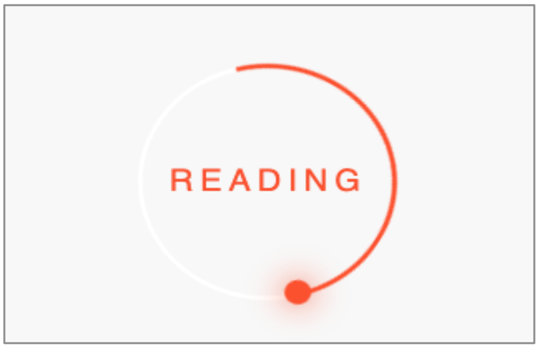

After the reader has completed this task, you get a new screen in which you see one of the pictures and a overimposed grid:

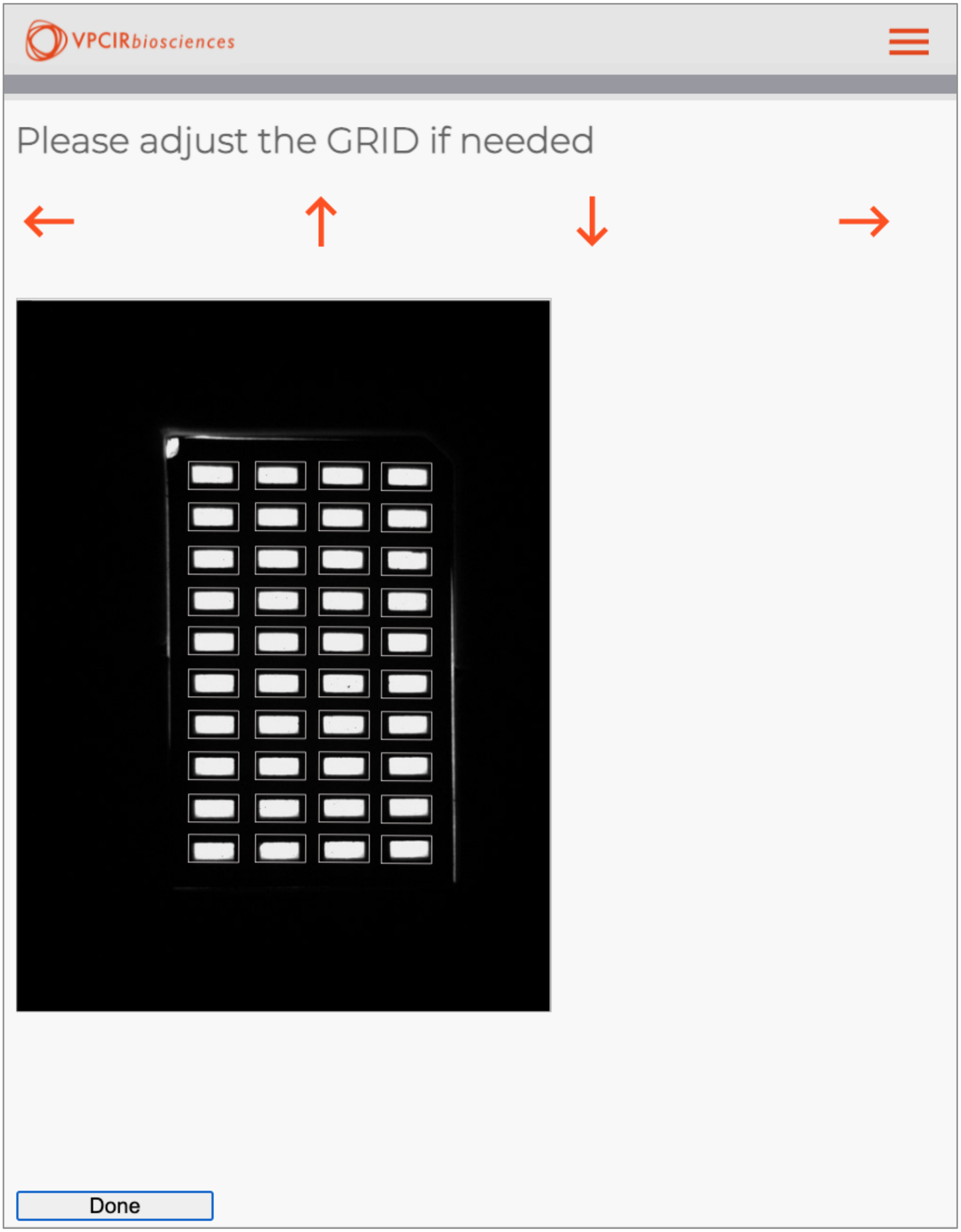
VPCIRbiosciences
Please adjust the GRID if needed
Done

In this screen you should adjust the grid to the wells by means of moving the grid up and down and left and right with arrow buttons. The area inside each rectangle of the grid you can move is the area that will be used in the calculation for the final result that you obtained.

After moving the grid, if necessary, you will press the button “Done” and then you should wait until the reader makes the appropriate calculations.

After these calculations are completed,you are directed to the View / Edit read screen where you can change the name and comments of the read, browse the frames and get a report anytime you want from a specific frame as has been explained above

Reports

After you click the reports option in the general menu you get the report submenu:

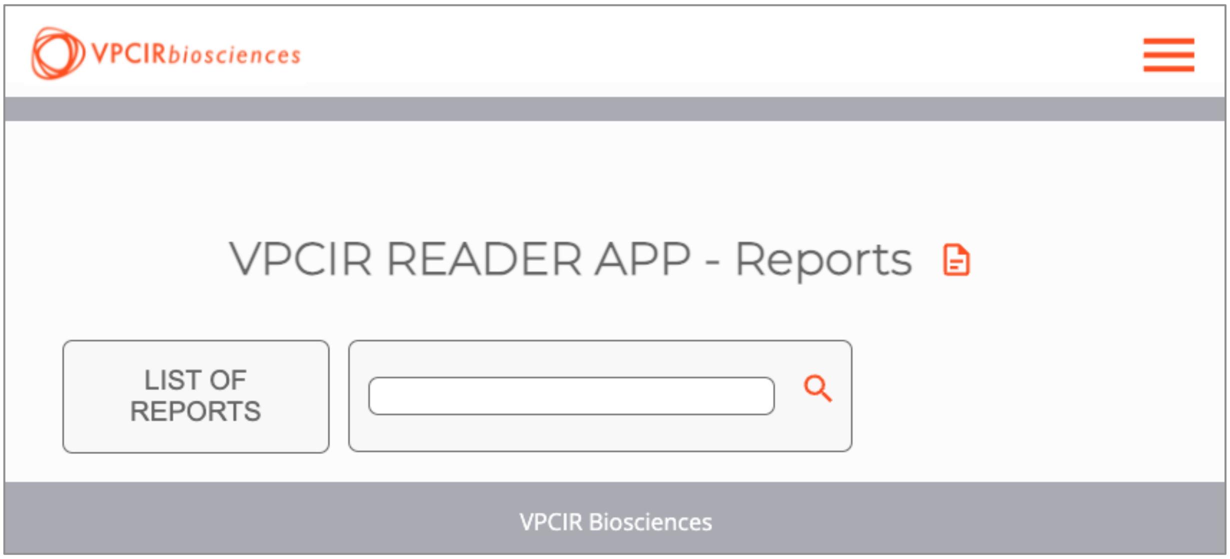

In this submenu you have the search and the List option as usual. In this case There is no “New Report” option because the report corresponding to the current frame is created with a simple click from the View / Edit Read screen as explained above.

Listing Reports

When you search or click the LIST OF REPORTS button , you get a list of reports:

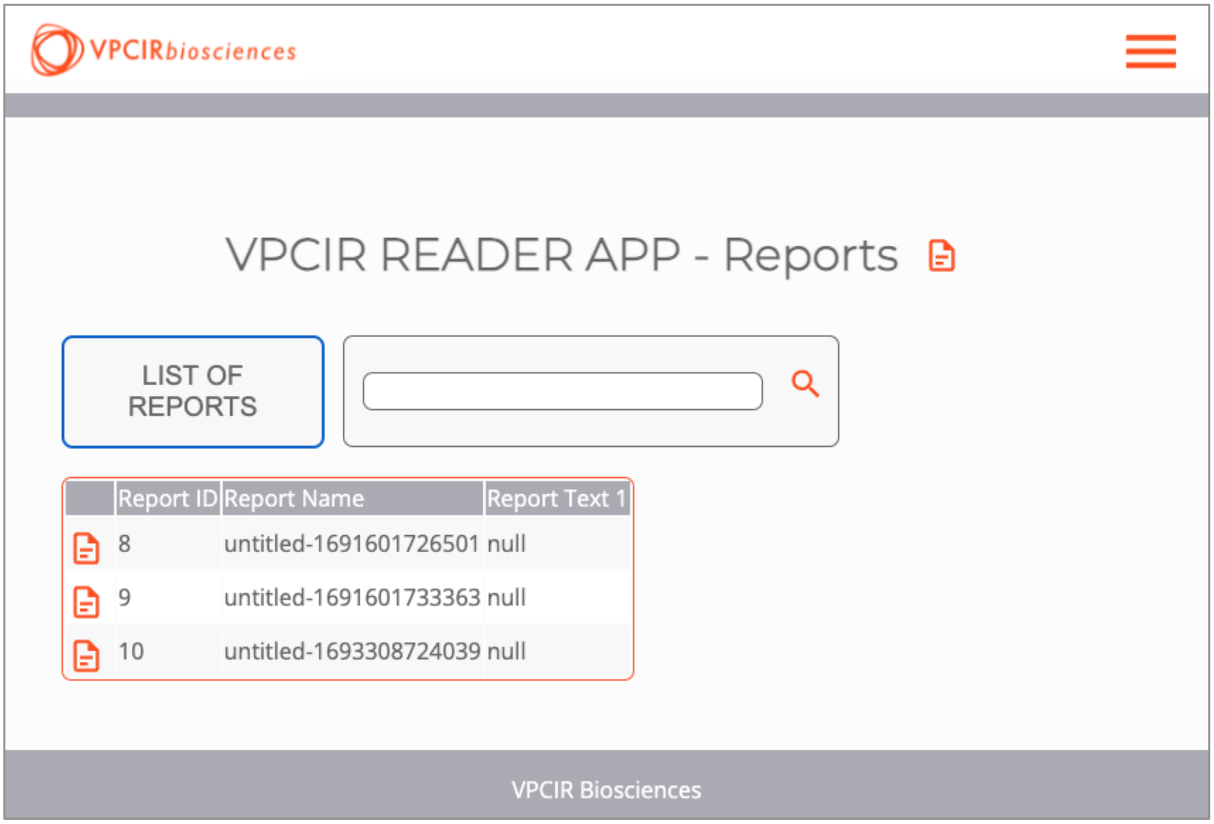

View / Edit Reports

If you click on the report (document) icon you get the view / edit report screen. The first part of the screen is the name and comments that are always editable and once change you shoul press the “enter” button:

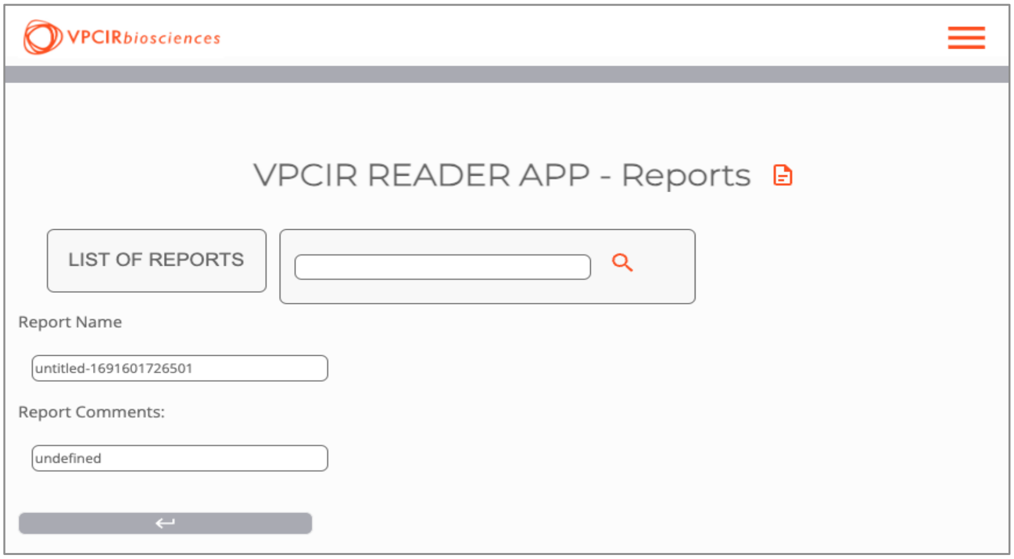

The next section of this screen has the actual picture of the frame from which the report was saved:

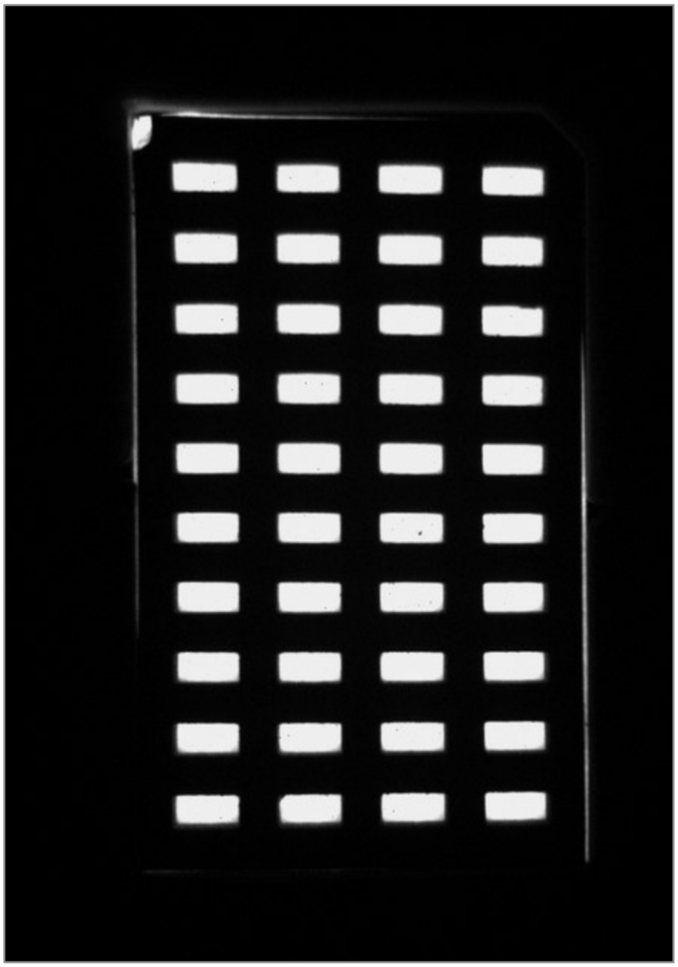

After this picture you can see the grid with information of sample, dilution and value of reading for each well:

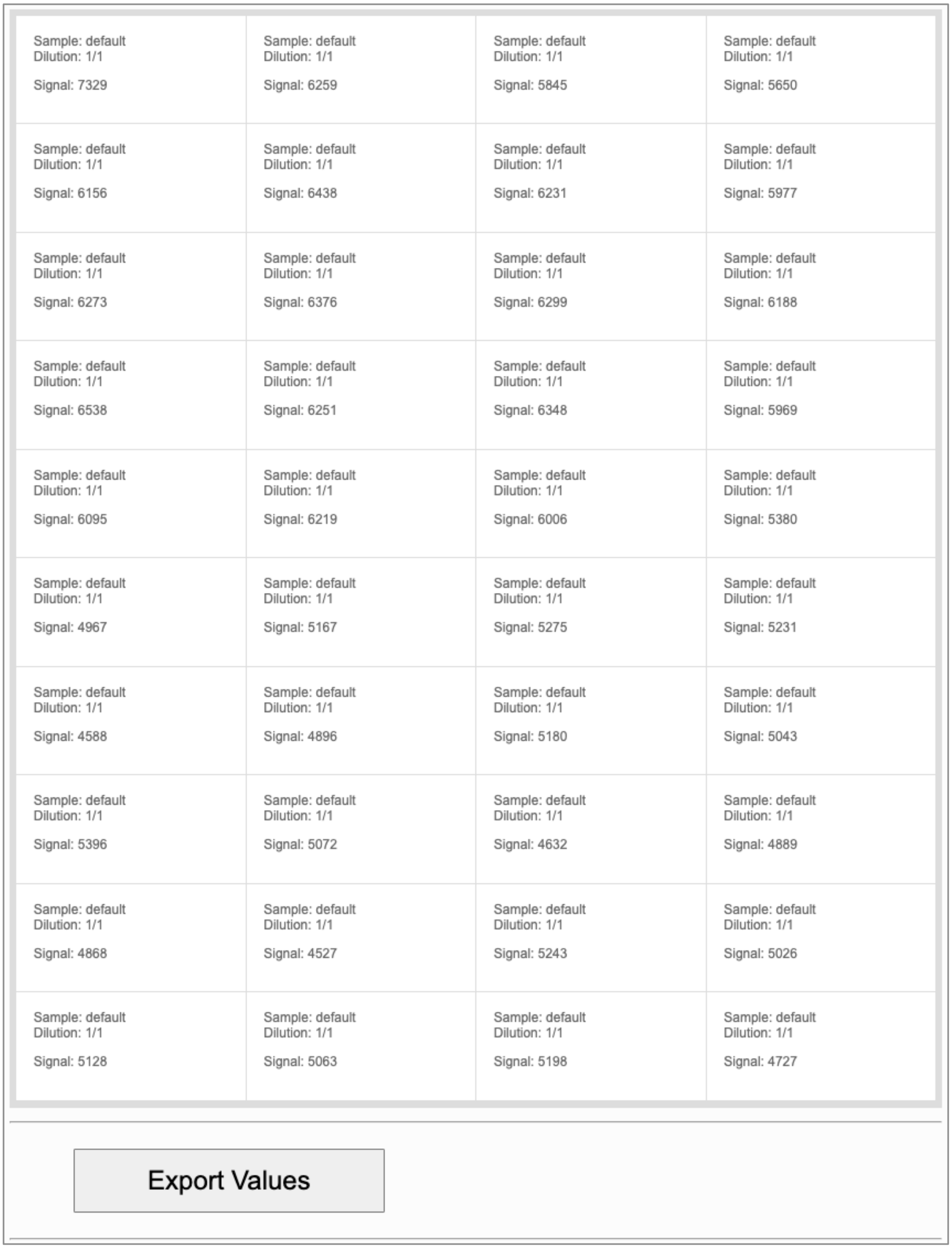

Sample: default<br>Dilution: 1/1<br>Signal: 7329	Sample: default<br>Dilution: 1/1<br>Signal: 6259	Sample: default<br>Dilution: 1/1<br>Signal: 5845	Sample: default<br>Dilution: 1/1<br>Signal: 5650
Sample: default<br>Dilution: 1/1<br>Signal: 6156	Sample: default<br>Dilution: 1/1<br>Signal: 6438	Sample: default<br>Dilution: 1/1<br>Signal: 6231	Sample: default<br>Dilution: 1/1<br>Signal: 5977
Sample: default<br>Dilution: 1/1<br>Signal: 6273	Sample: default<br>Dilution: 1/1<br>Signal: 6376	Sample: default<br>Dilution: 1/1<br>Signal: 6299	Sample: default<br>Dilution: 1/1<br>Signal: 6188
Sample: default<br>Dilution: 1/1<br>Signal: 6538	Sample: default<br>Dilution: 1/1<br>Signal: 6251	Sample: default<br>Dilution: 1/1<br>Signal: 6348	Sample: default<br>Dilution: 1/1<br>Signal: 5969
Sample: default<br>Dilution: 1/1<br>Signal: 6095	Sample: default<br>Dilution: 1/1<br>Signal: 6219	Sample: default<br>Dilution: 1/1<br>Signal: 6006	Sample: default<br>Dilution: 1/1<br>Signal: 5380
Sample: default<br>Dilution: 1/1<br>Signal: 4967	Sample: default<br>Dilution: 1/1<br>Signal: 5167	Sample: default<br>Dilution: 1/1<br>Signal: 5275	Sample: default<br>Dilution: 1/1<br>Signal: 5231
Sample: default<br>Dilution: 1/1<br>Signal: 4588	Sample: default<br>Dilution: 1/1<br>Signal: 4896	Sample: default<br>Dilution: 1/1<br>Signal: 5180	Sample: default<br>Dilution: 1/1<br>Signal: 5043
Sample: default<br>Dilution: 1/1<br>Signal: 5396	Sample: default<br>Dilution: 1/1<br>Signal: 5072	Sample: default<br>Dilution: 1/1<br>Signal: 4632	Sample: default<br>Dilution: 1/1<br>Signal: 4889
Sample: default<br>Dilution: 1/1<br>Signal: 4868	Sample: default<br>Dilution: 1/1<br>Signal: 4527	Sample: default<br>Dilution: 1/1<br>Signal: 5243	Sample: default<br>Dilution: 1/1<br>Signal: 5026
Sample: default<br>Dilution: 1/1<br>Signal: 5128	Sample: default<br>Dilution: 1/1<br>Signal: 5063	Sample: default<br>Dilution: 1/1<br>Signal: 5198	Sample: default<br>Dilution: 1/1<br>Signal: 4727

At the bottom of the screen you have an “Export Values” button that, if pressed, opens a new tab in you browser with the results shown in a convenient way to copy and paste in an application like excel as is text separated by “,” :

```
Report Name:,untitled-1691601726501
From Read Frame Number:,7
From Read ID:,6
From Read Name:,reading de un experimento hecho con copia
From experiment ID:,10
From Experiment Name:,copia para probar reading
Assay Type Name:,c4 10r assay
Samples and Values:
Sample Name,Dilution,Value
default,1/1,7329
default,1/1,6259
default,1/1,5845
default,1/1,5650
default,1/1,6156
default,1/1,6438
default,1/1,6231
default,1/1,5977
default,1/1,6273
default,1/1,6376
default,1/1,6299
default,1/1,6188
default,1/1,6538
default,1/1,6251
default,1/1,6348
default,1/1,5969
default,1/1,6095
default,1/1,6219
default,1/1,6006
default,1/1,5380
default,1/1,4967
default,1/1,5167
default,1/1,5275
default,1/1,5231
default,1/1,4588
default,1/1,4896
default,1/1,5180
default,1/1,5043
default,1/1,5396
default,1/1,5072
default,1/1,4632
default,1/1,4889
default,1/1,4868
default,1/1,4527
default,1/1,5243
default,1/1,5026
default,1/1,5128
default,1/1,5063
default,1/1,5198
default,1/1,4727
```
